# Nurse staffing models that rely on employment of temporary nurses: A realist review

**DOI:** 10.1016/j.ijnsa.2026.100537

**Published:** 2026-04-28

**Authors:** Inge Wolbers, Catharina van Oostveen, Dewi Stalpers, Greta G. Cummings, Kaitlyn Tate, Lisette Schoonhoven, Pieterbas Lalleman, ResoNANCE consortium

**Affiliations:** a University of Applied Sciences Utrecht. Heidelberglaan 7, Utrecht, CS 3584, the Netherlands; b Erasmus School for Health Policy and Management, section Health Services Management and Organisation, Erasmus University Rotterdam, Burgemeester Oudlaan 50, Rotterdam, PA 3062, the Netherlands; c Dept of General Practice and Nursing Science, Julius Center for Health Sciences and Primary Care, University Medical Center Utrecht, Utrecht University, Heidelberglaan 100, Utrecht, CX 3584, the Netherlands; d Faculty of Nursing, College of Health Sciences, University of Alberta, Dianne and Irving Kipnes Health Institute 11405 - 87 Ave NW, Edmonton, AB T6G 1C9, Canada; e Fontys University of Applied Sciences Rachelsmolen 1, Eindhoven, 5612MA, the Netherlands

**Keywords:** Nurse staffing, Temporary nurses, Realist review, Situated knowledge

## Abstract

**Background:**

Nurse shortages are a global issue. Adequate nurse staffing is considered crucial for the quality and safety of care. To efficiently address demands for care, healthcare organisations often employ temporary nursing staff to maintain adequate staffing levels. However, evidence concerning the association between the use of temporary staffing and patient and nurse outcomes is mixed. Work environment, patient needs, nurses' characteristics, and their perspectives on best care practices influence the match between care demand and supply, ultimately leading to different outcomes.

**Objective:**

As part of a multi-phase realist review, we aimed to test an initial programme theory on nurse staffing models that frequently use temporary nurses unfamiliar with the unit to determine through which mechanisms these models produce patient, staff, and health system outcomes in particular contexts.

**Methods:**

We systematically searched for empirical evidence on nurse staffing models that frequently used temporary nurses. After extracting context-mechanism-outcome configurations from the empirical evidence, we categorised data as supporting, explaining, or refuting our initial theory and synthesized findings to generate a refined and rival programme theory. To augment and refine our programme theory, we conducted a realist focus group and an interview with purposively selected managers and directors from care organisations and agencies, as well as a temporary nurse.

**Results:**

We included 49 studies in our analysis that met thresholds of relevance, richness, and rigour. Findings partially supported the initial programme theory, which proposed that heavy reliance on temporary nurses unfamiliar with their assigned units could threaten care continuity, overburden permanent staff, and lead to adverse outcomes. However, this held particularly when staffing levels were low at baseline. A rival programme theory emerged, showing the advantages of using temporary nurses if there is a supportive work environment. Both theories underscore the importance of situated, often tacit knowledge, held by permanent nurses that influences staffing success.

**Conclusions:**

With temporary nurses a continuing staffing presence, we have highlighted the need for more research on nurse staffing models that integrate both permanent and temporary nurses. Because care organizations must ensure adequate nursing staff levels, they should consider focusing on strengthening collaboration by creating supportive work environments that are fuelled with situational knowledge. Key strategies may include fast onboarding and orientation for temporary nurses to familiarise them with patients and contexts. In intuitive work environments, temporary nurses may be able to utilise their experience and expertise, consequently enabling mutual learning.

**What is already known**

• Temporary nurses are frequently employed to maintain staffing levels, yet evidence on their impact on patient and nurse outcomes is mixed.
• The employment of temporary nurses only partly mitigates the negative association between low staffing levels and adverse patient outcomes.
• The nursing work environment consistently emerges as a key contextual factor influencing both the employment of temporary nurses and the quality of care.

**What this paper adds**

• We have provided an explanatory theory of how the deployment of temporary nurses may influence outcomes in terms of a context-mechanism-outcome configuration.
• Current work environments may be often insufficiently equipped to enable experienced but unfamiliar temporary nurses to work effectively and intuitively.
• We have noted the potential importance of intuitive work environments for temporary nurses’ access to situational knowledge.

Alt-text: Unlabelled box dummy alt text

## 1 Background

Nurse staffing levels and skill mix are essential for care quality and safety (McHugh et al., 2021; Jutkowitz et al., 2023) and nurses' well-being (Shin et al., 2018). Global nursing shortages increasingly challenge the alignment of care demands with available resources, putting pressure on healthcare systems and patient safety (Buchan et al., 2022; Dutch Ministry of Health, Welfare and Sport, 2023). To address staffing challenges, healthcare organisations frequently employ temporary nurses, including internal staff (e.g., float or pool nurses), and external staff, such as per diem, travel, or agency nurses (adapted from Aiken et al., 2007). The duration of the temporary nurses also varies widely, from single shifts to extended placements.

The employment of temporary nurses reflects a policy logic of ‘governing by numbers’ (Porter, 1995; Wallenburg et al., 2023) aimed at maintaining continuity of care by alleviating permanent nurses’ workload. However, this policy strategy affects nursing work practice. Given the heterogeneity of temporary nurses and work environments, it remains unclear whether temporary nurses can effectively address supply-demand gaps or, instead, introduce additional pressures for patients, staff, and health systems.

Empirical findings on the impact of temporary nurses show nuanced, conditional effects on the quality of care. Across studies, researchers have found that higher proportions of temporary nurses may be associated with lower quality of care (Bae et al., 2010; Dall’Ora et al., 2020; Castle, 2009b), while lower proportions of temporary nurses may be associated with fewer safety incidents and, therefore, better quality (Jomaa et al., 2022; Bae et al., 2014a; Bae et al., 2010), particularly when related to low staffing levels (Griffiths et al., 2024; Senek et al., 2020; Saville et al., 2021). The effects of temporary nurse deployment are neither inherently positive nor negative but depend on how nurse staffing models interact with contextual factors, such as the work environment (Aiken et al., 2013; Bae et al., 2014b; Baker et al., 2019). However, the underlying mechanisms through which the use of temporary nurses generates these divergent outcomes remain poorly understood. From a realist perspective, examining how specific contexts trigger underlying mechanisms is essential to explaining this variation. Therefore, as part of a multi-phase realist review (Tate et al., 2024), we aimed to test an initial programme theory on staffing models that frequently use temporary nurses unfamiliar with the unit to identify the mechanisms through which these models generate patient, staff, and health system outcomes in particular contexts.

## 2 Method

### 2.1 Research design

A realist review aims to determine *what works, for whom, in what circumstances, how, and why* by expanding the traditional causal model, which assumes that intervention X leads to a given outcome (O) (Pawson et al., 2005). Additionally, realism explores the contexts (C) and mechanisms (M) that enable X to lead to O, transforming the X-O model into a context-mechanism-outcome model. This approach makes the realist review process contextually sensitive and explanatory, which is necessary when a topic is undertheorised or the evidence reveals nuanced and conditional effects, as in the employment of temporary nurses (Pawson et al., 2005). Underpinning this methodology is realist philosophy, which assumes that reality exists independently of our knowledge and can therefore exist at multiple layers within reality, from observable to latent, also known as ontological depth (Jagosh, 2019). This implies that when a phenomenon manifests, it is likely driven by mechanisms operating at different layers, among others, on individual, relational, community, and societal layers. This realist review was undertaken in iterative phases with an international team of researchers and decision-makers from Canada, Australia, Great Britain, and the Netherlands. See Tate et al. (2024) for a detailed description of the study protocol. During the first phase, initial candidate programme theories were identified through a literature review and focus groups to augment, refine, and prioritize initial programme theories. An initial programme theory is an explanatory framework that articulates how, why, for whom, and in what circumstances the phenomenon of interest is thought to work, by describing the context, the underlying mechanisms, and the outcomes. They are heuristic tools that help guide literature searching and data extraction. One expects them to evolve as one engages with the evidence —becoming more nuanced, identifying additional context-mechanism-outcomes configurations and recognizing how context affects mechanisms that generate outcomes (Pawson et al., 2005). It can be seen as the groundwork for the subsequent realist review by identifying concepts, theories, and potential underlying mechanisms. See Tate et al. (2025) for the identification and prioritisation of initial program theories.

The Dutch team adopted the following initial programme theory for further exploration and testing: a nurse staffing model that frequently uses non-full-time and temporary nurses who are unfamiliar with a unit [context], threatens care continuity, and permanent registered nurse (RN) staff will feel overburdened and demotivated [mechanisms], leading to poor teamwork, burnout, more adverse events, longer hospital stays, and increased costs [outcomes] (Tate et al., 2025).

### 2.2 Search methods

Structured searches were constructed to test and refine the initial programme theory. A purposive search strategy was developed with the assistance of an academic librarian to search for articles that evaluated staffing models focusing on the use of temporary nurses. The strategy was subsequently reviewed by an expert in library science and realist methods to ensure its appropriateness and accuracy. The search was conducted using MEDLINE, CINAHL, EMBASE, and Scopus databases. Moreover, a CLUSTER search was performed, containing **C**itation tracking, tracing **L**ead authors, Google **S**cholar searching, **U**npublished materials, **T**heory tracking, ancestry searching for **E**arly examples, and follow-up of **R**elated projects as the review progressed (Booth et al., 2013b). Various search terms for temporary nurses were used, such as bank nurse, part-time nurse, casual nurse, temporary nurse, agency nurse, per diem nurse, supplemental nurse, floating nurse, and travel nurse. The search was limited to records written in English or Dutch, and the initial search was conducted on May 31, 2023. The citation tracking was performed on October 14, 2024. The search string is presented in Supplementary Material File 1.

### 2.3 Selection procedure

Rayyan software (Ouzzani et al., 2016) was used to organise the realist review process. Records were screened in two stages by (IW, CO, DS & PL): title and abstract screening (IW & DS) and full-text screening (IW, CO, DS & PL). Studies with a title and abstract that matched our inclusion criteria were selected for full-text screening. Two researchers, blinded to each other’s decisions, screened the studies. Consensus meetings were held to resolve any conflict. A third researcher resolved conflicting decisions. The inclusion criteria consisted of two parts: the criteria in part A were used in all parts of the multi-phase realist review (Tate et al., 2024). Records had to include criteria 1 and 2, plus either 3 or 4, or 5 or 6. The inclusion criteria were: 1) a nurse staffing model (which may include other healthcare professionals) conceptualised, developed, implemented, or evaluated in any of the following health settings (in-patient or out-patient settings, including acute care settings [emergency departments and in hospital], primary care settings, community care settings, palliative care settings, transitional or rehabilitation care settings, and continuing care settings). 2) A nurse staffing model conceptualised, developed, implemented, or evaluated in high-income countries based on World Bank income groups (https://data.worldbank.org/income-level/high-income). 3) A formal or substantive theory, mid-range theory, or theoretical/conceptual framework that describes how nurse staffing models are intended to work. 4) Ideas about how nurse staffing models are intended to work or a critique of the ideas underlying how nurse staffing models are intended to work. 5) Stakeholder accounts or opinions of how nurse staffing models do, or do not, work. 6) Outline, discuss or review of potential unintended consequences of nurse staffing models. Additionally, Part B was applied to our adopted initial programme theory: empirical evidence that is relevant, rich, and robust and supports, challenges, or gives insight into a nurse staffing model. In this case, the model that frequently uses non-fulltime and temporary nurses who are unfamiliar with a unit (context) threatens care continuity, and permanent RN staff will feel overburdened and demotivated (mechanisms), leading to poor teamwork, burnout, more adverse events, longer hospital stays, and increased costs (outcomes). The full inclusion and exclusion criteria are presented in Supplementary Material File 2.

### 2.4 Quality appraisal

We assessed quality based on whether and to what extent the data contributed to theory building and testing. Quality appraisal was based on three key criteria: relevance, richness, and rigour (Dada et al., 2023). Relevance refers to whether the data can contribute to theory building or testing for the included contexts or regions. Richness involves the degree of theoretical thickness and conceptual richness, explaining how an intervention is expected to work based on grounded and detailed descriptions. Rigour focuses on the trustworthiness of the source and the coherence and transparency of the theory (Dada et al., 2023; Tate et al., 2024) to explore confirmatory and contradictory findings in relation to our initial programme theory (Booth et al., 2013a). The relevance, richness, and rigour were assessed with questions adapted from Jagosh et al. (2011) to determine the richness of description for nurse staffing models and completeness, combined with the ‘traffic light system’ method proposed by Morton et al. (2021). Records were consequently appraised for minor (green), moderate (yellow), or major (red) concerns regarding relevance, richness, and rigour. Consensus meetings were held with two researchers (IW and CO or DS, or PL) to discuss insights and judgments and reach a joint appraisal. To ensure consistency, one researcher (IW) was involved in all decisions. The quality appraisal instrument used, the procedure for application, and an example are presented in Supplementary Material File 3.

### 2.5 Data extraction and synthesis

Data extraction and synthesis were reached in three stages. In the first stage, we worked on consistency across the multinational team. First of all, a meeting among the methodologists from the multinational team was held to establish a shared understanding of terms, concepts, and processes required for extraction. We also agreed that at least three independent reviewers would be part of the review team. In the Netherlands, we chose three independent reviewers (CO, DS, PL) and one PhD candidate (IW). Second, a small set of initial extractions was sent to one of the methodologists and was cross-checked to confirm a common interpretation. Thereafter, two researchers independently extracted information using a standardised data extraction form (Tate et al., 2024). Data were extracted according to key realist components of context, mechanisms, and outcomes. Contexts include social, economic, and political structures; healthcare settings or social conditions, programme participants, programme staffing and experience; and geographical, cultural, and historical contexts (Tate et al., 2024; Pawson and Tilly, 1997). Mechanisms may not be directly observable but may be explored through theory building and testing (Tate et al., 2024). As cited in Wong et al. (2012, p.91), mechanisms can be described as *“underlying entities, processes, or (social) structures which operate in particular contexts to generate the outcomes of interest”*. After data extraction, consensus meetings were held among the researchers to verify the context-mechanism-outcome configurations and to address and resolve discrepancies through discussion. In the second stage, analysis and synthesis were undertaken to augment and refine the programme theory. The initial programme theory guided development and refinement throughout the process. Extracted contexts, mechanisms, and outcomes were subsequently combined into context-mechanism-outcome configurations. These were arranged vertically in an Excel set and then compared, merged, collapsed, and refined. We repeatedly returned to the primary source and the data extraction form to ensure consistency with the original intent. A written narrative was then developed to examine how the configurations supported, refined, or refuted the initial programme theory. Configurations that were unaligned with or refuted the initial programme theory informed the rival programme theory. Regular meetings with Dutch team members supported theory development and further refinement. The tested and rival programme theories were subsequently discussed with the Dutch team and the international consortium, leading to additional refinement. In the third stage, after synthesising the preliminary results, a realist focus group was conducted to examine whether and how both theories were reflected in practice (Malengreaux et al., 2025), further validating and refining the theories. We purposively sampled four managers and directors from healthcare organisations across varied settings, as well as two directors from staffing agencies to ensure maximum variation and dispersion (Palinkas et al., 2015). Participation was contingent upon involvement in the employment of temporary nurses. Finally, an interview with an agency nurse was conducted to validate and refine the findings from a nursing perspective. The study was approved by the Ethical Committee for Research of the University of Applied Sciences Utrecht (reference number 149–000–2021).

## 3 Results

### 3.1 Search outcome

**Fig. 1:**
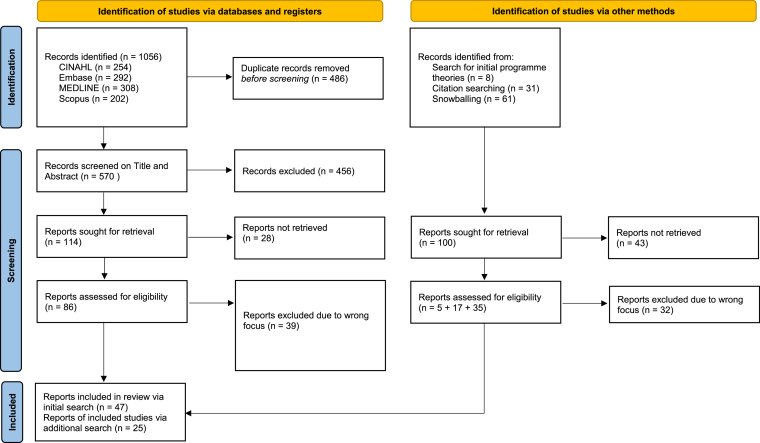
PRISMA flow diagram. This work is licensed under CC BY 4.0. To view a copy of this license, visit https://creativecommons.org/licenses/by/4.0/.

Source: Page MJ, et al. BMJ 2021;372:n71. doi: 10.1136/bmj.n71.

The initial search identified a total of 1056 records. After duplicates were removed, 570 titles and abstracts were screened, and 456 records were excluded because of irrelevance to the subject. In total, 86 records were assessed for eligibility. In addition, identification of studies via the search for initial programme theories, a citation search, and snowballing resulted in 25 additional records. In total, 72 records were included for quality assessment (Fig. 1).

### 3.2 Study characteristics

Of the included records (Table 1), 56 were original research, 11 were secondary research, one was an opinion paper (Manthey, 2001), and four were descriptions of practices. Most records originated from the United States of America (USA) (*n* = 41) and the United Kingdom (UK) (*n* = 13). The majority of the records described the use of temporary nurses in hospitals (*n* = 45) or a nursing home (*n* = 9). The included records were published between 1989 and 2024.

**Table 1:** Overview of selected studies with study descriptors combined with quality appraisal.

Author(s); (year of publication); country	Setting	Resource type	Design	Objective	Quality appraisal concerns	Taken into analysis
Aiken et al. (2007); USA	Hospital	Original research	Observational, cross-sectional	To promote evidence-based decision making regarding hospital staffing, the authors examined the characteristics of supplemental nurses, as well as the relationship of supplemental staff to nurse outcomes and adverse events.	Minor	Yes
Aiken et al. (2013); USA	Hospital	Original research	Observational, cross-sectional	To determine the association between the use of agency-employed supplemental registered nurses to staff hospitals and patient mortality and failure to rescue	Minor	Yes
Alvarez et al. (2011); USA	Hospital	Original research	Observational, longitudinal	To discuss the outcomes of their survey of LTACH chief nursing officers that demonstrated LTACH quality indicators and overall patient satisfaction were within nationally accepted benchmarks even with higher levels of outsourced nurses used in this post-acute care setting.	Major	No
Anderson et al. (1996); USA	Army Medical Center	Original research	Descriptive	To describe the perceived impact of supplemental agency nurses upon the quality of patient care, unit cohesiveness, and morale of permanently assigned personnel at an army medical center.	Major	No
Bae et al. (2010); USA	Hospital	Secundary research	Observational, longitudinal	To investigate the association between use of temporary nurses and nurse (needlestick and back injuries) and patient (patient falls and medication errors) safety outcomes at the nursing unit level.	Minor	Yes
Bae et al. (2014a); USA	Hospital	Original research	Observational, longitudinal	To examine the nature and prevalence of the use of temporary nursing staff in intensive care units and relationships between the use of temporary nursing staff and the occurrence of nosocomial infections.	Minor	Yes
Bae et al. (2014b); USA	Hospital	Original research	Observational, longitudinal	To examine the relationship of nurse staffing to quality of patient care outcomes, by including not only nursing turnover and temporary nursing staff but also nurse staffing levels and registered nurse skill mix.	Minor	Yes
Bajorek & Guest (2019); UK	Acute care	Original research	Descriptive	To explore the impact of temporary workers on the perceptions, attitudes and behaviour of permanent staff with particular reference to their implications for patient safety and service quality in hospital accident and emergency departments.	Minor	Yes
Batch et al. (2009); Australia	Hospital	Secondary research	Literature review	To explore the relationship between the increasing casualisation of the nursing workforce and, among other things, the communication practices of nurses within healthcare organisations.	Major	No
Batch and Windsor, (2015); Australia	Hospital	Original research	Qualitative	To explore the relationship between nursing casualisation and the culture of communication for nurses in a healthcare facility.	Minor	Yes

Author(s); (year of publication); country	Setting	Resource type	Design	Objective	Quality appraisal concerns	Taken into analysis
Baker et al. (2019); UK	Mental health care	Original research	Qualitative	To explore the impact of staffing and skill mix on safety and quality of care in mental health inpatient and community services.	Minor	Yes
Bellon (2023); USA	Hospital	Practice	-	To demonstrate how Advocate Aurora Health successfully established an internal staffing agency to recruit and retain nursing staff *(not explicitly stated by the author).*	Major	No
Berg Jansson & Engström (2017); Sweden	Hospital	Original research	Qualitative	To describe critical care nurses' experiences of working with or as temporary agency staff.	Moderate	Yes
Birmingham et al. (2019); Australia	Hospital	Secondary research	Integrative literature review	To examine experienced RNs' motivations for choosing agency work, their experiences and perceptions of agency nursing, and how they meet their regulatory professional development obligations.	Minor	Yes
Bloom et al. (1997); USA	Hospital	Original research	Observational, cross-sectional	To assess the effects of four nurse staffing strategies on the efficiency of patient care delivery.	Major	No
Bourbonniere et al. (2006); USA	Nursing home	Original research	Observational, longitudinal	To characterise the extent to which nursing homes use contract RNs and LPNs, to determine what kinds of nursing home market situations foster the use of contract RNs and LPNs, and to relate use of contract nursing staff to the quality of care in nursing homes indicated by health deficiency citations.	Moderate	Yes
Brooks (2024); USA	Hospital	Practice	-	None stated	Major	No
Castle & Engberg (2007); USA	Nursing home	Original research	Observational, cross-sectional	To examine the influence of staffing levels, turnover, worker stability and agency staff on quality of care in nursing homes.	Minor	Yes
Castle & Engberg (2008a); USA	Nursing home	Original research	Observational, cross-sectional	The aim of this research was not to disprove the notion that more staffing is better, but rather to show the limitations of using this simple headcount (i.e., using staffing levels) when examining quality.	Minor	Yes
Castle & Engberg (2008b); USA	Nursing home	Original research	Observational, cross-sectional	To examine the influence that agency staff has on quality of care in a large sample of nursing homes	Moderate	Yes
Castle et al. (2008); USA	Nursing home	Original research	Observational, cross-sectional	To examine the relationship between NA agency staffing levels and quality of care in a large sample of nursing homes.	Minor	Yes
Castle (2009a); USA	Nursing home	Original research	Observational	To examine the advantages and disadvantages of using agency staff	Moderate	Yes
Castle (2009b); USA	Nursing home	Original research	Observational, cross-sectional	To describe the extent and degree of use of agency staff by nursing homes and examine the relationship between agency staffing levels and quality of care in a large sample of nursing homes.	Moderate	Yes
Dall’Ora et al. (2020); UK	Hospital	Original research	Observational, longitudinal	To explore the association between the levels of temporary nurse staffing and patient mortality.	Minor	Yes
Djukanovic et al. (2022); Sweden	Hospital	Original research	Qualitative	To describe continuity from the perspective of working as an agency nurse.	Moderate	Yes

Author(s); (year of publication); country	Setting	Resource type	Design	Objective	Quality appraisal concerns	Taken into analysis
Dziuba-Ellis (2006); Canada	-	Secondary research	Literature review	This review of the literature explores those aspects of float pools and resource teams and differentiates between these two staffing models.	Major	No
Fagefors et al. (2022); Sweden	Hospital	Original research	Mixed-methods	To explore the practical barriers for staff pooling in a healthcare system as managers perceive them	Moderate	Yes
Faller et al. (2018); USA	Hospital	Original research	Observational, longitudinal	This study develops a more complete and accurate portrayal of the return on investment for travel nurse utilization by examining a hospital’s full costs for core staffing, not just the pay rate component for regular hours	Major	No
Fasano et al. (2023); USA	Hospital	Practice	-	This paper describes the development and outcomes associated with an innovative approach in addressing contemporary staffing constraints through the development of a centralised, flexible, and resilient new ‘graduate nursing resource team’ in an academic health system.	Major	No
Ferguson et al. (2020); USA	Hospital	Original research	Observational, cross-sectional	To explore the relationship between the use of contract nurses and two key nurse-sensitive outcomes, HAPIs and falls.	Moderate	Yes
FitzGerald and Bonner (2007); Australia	Hospital	Original research	Qualitative	To identify the factors that enable and obstruct nurses working with the casual pool to deliver high standards of nursing care within a multi-disciplinary team.	Major	No
Forster et al. (2006); Australia	Hospital	Original research	Mixed-methods	To describe the impact of staffing issues on the provision of quality postnatal care from the perspective of care providers.	Major	No
Gahrmann and Klumb, (2024); Switzerland	Hospital	Original research	Observational, cross-sectional	To explore illegitimate tasks as a potential mechanism that links permanent nurses' perceived exposure to temporary nurses to lower levels of affective organizational commitment.	Moderate	Yes
Gan (2022); USA	Hospital	Original research	Scoping review	To provide an overview of the causes and consequences of envy among clinical nurses.	Moderate	Yes
Gan (2024); USA	Hospital	Original research	Qualitative	To discuss how participants’ work arrangement enabled them to organize their work and nonwork lives in ways that were subjectively meaningful to them; (2) highlights how participants gave meaning to their work in relation to their social ties; and (3) contributes to the communication literature on meanings of work and human flourishing in communication studies.	Moderate	Yes
Gnanlet et al. (2023); USA	Hospital	Original research	Observational, longitudinal	To investigate the relationship between travel nurses and patient care to identify if consistent significant associations exist and how structural and process variables may influence such associations.	Moderate	Yes

Author(s); (year of publication); country	Setting	Resource type	Design	Objective	Quality appraisal concerns	Taken into analysis
Griffiths et al. (2021); UK	Hospital	Original research	Model-based simulation	To model the cost-effectiveness of different approaches to planning baseline numbers of nurses to roster on general medical/surgical units while using flexible staff to respond to fluctuating demand.	Minor	Yes
Griffiths et al. (2024); UK	Hospital	Original research	Observational, longitudinal	To explore the association of the composition of the nursing team with the risk of patient deaths	Minor	Yes
Hass et al. (2006); UK	ICU	Original research	Qualitative	To describe the lived experiences of the full-time agency nurses working in the environment of the intensive care unit.	Moderate	Yes
Hemann & Davidson (2012); USA	Ambulatory care setting	Secondary research	Literature review	*No objective described.*	Major	No
Hickey et al. (2024); USA	Hospital childcare	Original research	Qualitative	To explore the experience and perceived value of travel nurses in a children’s hospital	Major	No
Hughes & Marcantonio (1991); USA	Hospital	Original research	Observational	To examine recruitment-, retention-, and compensation-related differences between agency and hospital staff nurses.	Major	No
Hurst & Smith (2011); UK	Hospital	Original research	Observational, cross-sectional	To compare temporary and permanent work activity, cost and quality of care	Minor	Yes
Jamieson et al. (2008); Australia	Hospital	Original research	Qualitative	To develop a theory that explains the 'realities' of part-time nursing	Minor	Yes
Jomaa et al. (2022); Canada	Rehabilita-tion	Original research	Descriptive	To describe the organisation of nursing services (staffing, scope of practice, teamwork) and its association with medication errors and falls, in rehabilitation units	Minor	Yes
Jones et al. (2021); Australia	Primary Health Care	Original research	Observational, cohort	To evaluate the relationship between markers of staff employment stability and use of short-term healthcare workers with markers of quality of care.	Moderate	Yes
Kirby & Hurst (2014); UK	Community care	Practice	Observational, audit	To understand the current workload, the acuity and dependency of patients being cared for in the community by district nursing teams, by using an evidence-based tool.	Major	No
Krzyzewski et al. (2023); USA	-	Secondary research	Integrative review	To determine best practices for promoting safe patient care delivery by travelling clinical staff.	Minor	Yes
Lien, (2023); USA	Nursing home	Original research	Qualitative	To show how gig service, while empowering care suppliers, has led to the precarisation of care workers and care environments.	Minor	Yes
Ma et al. (2022); Australia	Residential care	Original research	Observational, longitudinal	To examine whether quality of care outcomes differ for Australian residential aged care facilities based on their reliance on agency care staff.	Minor	Yes
Manias et al. (2003); Australia	Hospital	Original research	Qualitative	To gain an in-depth understanding of agency nurses’ clinical practice, their relationships with the employing agency, hospitals and permanent nurses, and their professional status.	Moderate	Yes

Author(s); (year of publication); country	Setting	Resource type	Design	Objective	Quality appraisal concerns	Taken into analysis
Manthey (2001); USA	-	Opinion			Major	No
Massey et al. (2009); UK	Hospital	Original research	Descriptive, case study	To analyse bank and agency nursing staffing patterns and factors that impact on these patterns.	Major	No
McHugh (1997); USA	Hospital	Original research	Model-based simulation	To use computer simulation modelling to examine the costs and staffing outcomes of two different ‘float’ policies in a midwestern veterans administration hospital	Major	No
Oliveira et al. (2023); Switzerland	Psychiatry	Secondary research	Observational, cross-sectional	To investigate possible associations between the frequency of temporary nurse deployments and permanently employed nurses’ outcomes, including staffing levels in Swiss psychiatric hospitals.	Moderate	Yes
Oppel & Young (2018); USA	Hospital	Original research	Observational, cross-sectional	To examine the relationship between nurse staffing patterns and patients’ experience of care in hospitals with a particular focus on staffing flexibility.	Moderate	Yes
Owens et al. (2024); USA	Hospital	Original research	Qualitative	To explore factors related to a travel nurse completing or extending an assignment or leaving before the end of an assignment.	Moderate	Yes
Peerson et al. (2002); Australia	Hospital	Original research	Descriptive	To describe the professional relationship between hospitals and nursing agencies, utilisation trends of agency nurses, and institutional policies relating to the employment of agency nurses.	Moderate	Yes
Pham et al. (2011); USA	Hospital	Original research	Observational, cross-sectional	To evaluate whether temporary staff medication errors would be associated with more severe harm than permanent staff medication errors.	Moderate	Yes
Polancich et al. (2023); USA	Hospital	Original research	Observational, cross-sectional	The purpose of this study was to examine nurse staffing while describing the relationships that exist in staffing and quality associated with nursing care during the COVID-19 pandemic, a significantly challenging time for nurse staffing	Moderate	Yes
Pradhan et al. (2024); USA	Hospital	Original research	Observational, cross-sectional	To investigate the association of agency labour with hospital financial performance.	Moderate	Yes
Saville et al. (2020); UK	Hospital	Original research	Model-based simulation	Using computer simulation to estimate the costs and understaffing/overstaffing rates delivered/ caused by different approaches to setting staffing establishments.	Moderate	Yes
Senek et al. (2020); UK	Acute care	Secondary research	Observational, cross-sectional	To examine the occurrence of ‘care left undone’ understaffing and temporary staffing across acute sector settings.	Moderate	Yes
Simpson & Simpson (2019); UK	-	Secondary research	Scoping review	To perform a scoping review about why nurses continue to choose non-permanent nursing work.	Minor	Yes
Spector et al. (2024); USA	Hospital	Original research	Observational, cross-sectional	Do staff and travel nurses differ on burnout, job satisfaction, turnover intentions, and perceptions of work assignments?	Major	No
Twigg et al. (2016); Australia	Rural care setting	Original research	Qualitative	To explore staffing issues and the workload drivers influencing nursing activities in designated small rural hospitals of Western Australia.	Moderate	Yes

Author(s); (year of publication); country	Setting	Resource type	Design		Quality appraisal concerns	Taken into analysis
Vander Weerdt et al. (2023); USA	Acute care	Secondary research	Systematic review	To investigate the relationship between travel nurses and patient care to identify if consistent significant associations exist and how structural and process variables may influence such associations.	Minor	Yes
Xue et al. (2012a); USA	Hospital	Original research	Observational, longitudinal	To examine the relationship between use of supplemental RNs and patient outcomes.	Minor	Yes
Xue et al. (2012b); USA	Hospital	Secondary research	Observational, cross-sectional	To examine trends in supplemental nurse characteristics, including demographics, educational qualifications and work experience, with the goal of informing nursing workforce policy and development	Major	No
Xue et al. (2015); USA	Hospital	Original research	Observational, longitudinal	To compare the relative hourly nursing personnel costs per patient day between supplemental nurses and permanent nurses (including benefits and overtime worked by permanent nurses. To identify the cost-efficiency (inefficiency) threshold for nursing personnel costs with regard to using supplemental nurses compared with no use of supplemental nurses (use of only permanent nurses).	Minor	Yes
Weech-Maldonado et al. (2004); USA	Nursing home	Original research	Observational	To consider the direct and indirect effects of staffing (RN staffing mix and full-time RN staffing) on patient outcomes.	Moderate	Yes
Zaranko et al. (2022); UK	Hospital	Original research	Observational, longitudinal	To examine the impact of nursing team size and composition on inpatient hospital mortality.	Minor	Yes

Note: HAPI (Hospital-acquired pressure injury); LTACH (long term acute care hospitals); LPN (licensed practical nurse); NA (nurse assistant); RN (registered nurse); UK (United Kingdom) USA (United States of America).

### 3.3 Quality appraisal

Based on the quality appraisal, 52 of the included articles met thresholds of relevance, richness, and rigour (Table 2); most records (*n* = 26) were appraised ‘minor concerns on relevance or richness’ and 26 ‘moderate concerns on relevance or richness.’ In total, 20 records did not meet the thresholds for relevance, richness, and rigour and were therefore excluded from the final analysis. Furthermore, as our study team determined that part-time nurses were conceptually distinct from temporary nurses, we excluded three articles focusing on part-time nursing (Jamieson et al., 2008; Oppel and Young, 2018; Weech-Maldonado et al., 2004), resulting in 49 articles included for data extraction.

**Table 2:** Context-mechanism-outcome analysis of included studies.

	Included studies	Temporary nurse category	Data aggregation level*	Context	Mechanism	Outcome
1	Aiken et al., 2007	Non-permanent nurses	Organisation	If a nurse staffing model is used that makes higher use (10–15%) of non-permanent nurses,		It does not lead to safety and quality problems for nurses or their patients, or to poor job satisfaction among permanent nurses.
				If higher levels of non-permanent nurses are used,	Then, non-permanent and supplemental nurses compensate for nurse staffing deficiencies (or for the problem of resource adequacy)	This is associated with lower levels of quality problems in hospitals.
				If a staffing model uses higher proportions of non-permanent nurses,	Then, non-permanent nurses may have a weaker commitment than permanent nurses to their jobs,	Leading to permanent nurses who are more likely to indicate intentions to leave their jobs within a year.
2	Aiken et al., 2013	Supplemental Registered Nurses	Organisation	If a nurse staffing model is used with higher proportions of SRNs, and they take into account the quality of the nurse work environment.		Then the associations between higher use of SRNs and higher mortality and failure to rescue disappear.
				If a nurse staffing model is used with higher proportions of SRNs and the nursing work environment is considered poor,	Then, they may have problems recruiting and retaining staff	
3	Bae et al., 2010	Temporary nurses (external or internal)	Unit	If a nurse staffing model is used with 15% or more external temporary RN hours, or 15% or more internal temporary RN hours	Permanent and temporary nurses have to work together, but collaboration is primarily based on stable work relationships. In nursing units that heavily depend on temporary nurses, stable work relationships thus teamwork are less present.	This is associated with an increase in reporting patient falls, and an increased likelihood for nurses to report back injuries.
				If a nurse staffing model uses 5–15% external temporary RN hours	Temporary nurses may recognise and respect their lack of familiarity with the medication administration systems, and are therefore more careful and make fewer reported errors. Or temporary nurses may have a long-term contract on a unit and may become familiar with medication processes.	This is associated with a reduction of medication errors compared to nursing units that do not use temporary RNs.

	Included studies	Temporary nurse category	Data aggregation level	Context	Mechanism	Outcome
4	Bae et al., 2014a	Temporary nursing staff	Unit	If a nurse staffing model in an ICU uses temporary staff		Then this is not significantly related to nurse-sensitive outcomes such as CLABSI or VAP^.^
				If the score for staffing and resource adequacy (one of the work environment measures) increased by one point (with possible scores from 1–4)		Then the odds for CLABSI decreased by 95%, and for VAP by 79%.
5	Bae et al., 2014b	Temporary nursing staff	Unit	If a nurse staffing model is used in acute care units where greater than 0-<0.3 care hours per patient day is provided by temporary RN staff, (compared to no use of temporary RN staff),		This is associated with increased patient falls and falls with injuries.
6	Bajorek and Guest, 2019	Temporary staff	Unit	If a nurse staffing model in an Acute Emergency department uses temporary staff that is unfamiliar with the unit,	Then, permanent staff are required to provide guidance, supervision and feedback, which distracts permanent staff from their work, taking them away from patients and rushing activities.	This does not directly result in a major risk to patient safety, but it would be more likely to affect the service quality of patient care.
					Because of the unfamiliarity, temporary staff might work slowly, potentially delaying processes.	
					The assumption among clinical managers is that permanent staff would supervise temporary staff, and there seems to be a level of tolerance amongst permanent staff regarding their supervision and aiding team stability.	
					Temporary staff may view this shift as an opportunity to absolve themselves from responsibility and to do a minimum amount of work, leaving more for others.	
				If a nurse staffing model in an acute emergency department frequently uses temporary nurses who are familiar with a unit,	Temporary staff have already developed a working relationship, and permanent staff have confidence in the temporary staff’s ability to provide timely, accurate and efficient patient care.	

	Included studies	Temporary nurse category	Data aggregation level	Context	Mechanism	Outcome
7	Batch and Windsor, 2015	Workforce casualisation	Unit	If the current nursing workforce is seen from an emerging dualistic workforce perspective (with full-time nurses on the one hand, and part-time, casual, and itinerant nurses on the other hand),	Then, casual, part-time, and itinerant employees have traditionally been viewed as a peripheral workforce, and this has a disruptive effect on communication between all staff. Communication can be recognised as the organisational cement.	
					Then, communication behaviours and policies in organisations need to be addressed at both the micro and macro levels, with an increased focus on strategies to improve team building and collegiality between nurses at all levels to enhance understanding of the needs associated with the range of employment strategies.	
8	Baker et al., 2019	Temporary agency workers	Individual	If a nurse staffing model is considered understaffed, it relies on temporary agency workers. If understaffing persists over time, it can develop into chronic understaffing.	Then, there is a decline in knowledge and experience circulating in the team.	Leading to the addition of burden to permanent nurses, staff sickness and poor recruitment and retention. There are limited specific services available for patients.
					Then, the addition of bank of agency staff is adding to, rather than relieving the burden.	
				In turn, chronic understaffing can lead to ‘unsafestaffing’. If a nurse staffing model is considered unsafestaffed,	Then, this leads to staff burden such as high workloads, lack of care continuity in staff because of reliance on bank/agency staff, resulting in a lack of developing therapeutic relationships, resulting in a lack of care continuity.	Resulting in unfavorable outcomes such as waiting lists for patients; less effective treatment, and eventually in more completed suicides.
9	Berg Jansson and Engström, 2017	Agency critical care nurses (CNNs)	Individual level	If a nurse staffing model is used that frequently uses agency critical care nurses who are unfamiliar with the unit,	Then agency CCNs work first and foremost close to the patient, whereas regular CCNs work in both regular patient-related tasks and long-term tasks related to work environment, which leads to a heavier workload for regular staff.	

	Included studies	Temporary nurse category	Data aggregation level	Context	Mechanism	Outcome
					Then, written instructions and clear routines (made visible by regular CNNs) facilitate the work of agency CNNs. This would not work if the staff is exclusively agency CNNs.	
					Then the agency CNN can be seen as a kind of knowledge extender, a person who carries and distributes knowledge to different hospitals/ ICUs.	
					Then, when too many agency CNNs, it can lead to a situation in which development ceases because the agency nurses are not part of the regular routines and processes in the ICU, so they are unclear about the goals of the ICU.	
10	Birmingham et al., 2019	Agency/ temporary/supplemental registered nurses	Individual	If a nurse staffing model is used that uses experienced agency nurses who are unfamiliar with an acute care unit, agency nurses should be allocated to appropriate areas where the nurses’ competencies and skills meet the wards’ requirements. If not, an agency nurse is asked to work outside their scope of practice.	Then, limited orientation at the commencement of employment in the specific clinical area reduced agency RNs' self-confidence (especially in layout and equipment).	This may affect the prompt and effective delivery of patient care
					Then, agency nurses may rely on the hospital nursing staff to provide relevant clinical information on allocated patients to assist in the planning and delivery of care. Hence, communication of patient care information is essential between hospital nursing staff and the experienced agency RN	
					Then, communication is essential to strengthen teamwork in any clinical environment. It can assist in the development of effective work relationships between permanent and temporary nurses (and all members of the nursing workforce).	

	Included studies	Temporary nurse category	Data aggregation level	Context	Mechanism	Outcome
					Then, less emotional support is provided to an agency RN, resulting in a ‘lack of belonging’.	
					Then the experienced RN agency nurse was expected to continue to deliver the patient care, whilst the permanent nurses attended the session.	
11	Bourbonniere et al., 2006	Contract nurses (RN and LPN)	Organisation	If a nursing home uses a staffing model that uses more temporary staff (RNs and LPNs),		This is associated with poor quality.
12	Castle and Engberg, 2007	Agency nurses	Organisation	If a nursing home uses higher rates of agency nurses (NAs, LPNs, and RNs)		This is associated with lower overall quality.
13	Castle and Engberg, 2008a	Agency nurses	Organisation	If a nurse staffing model in a nursing home is used where they take into account multiple staffing characteristics, such as staffing levels, agency staffing, stability, and professional staff mix,	Then it influences quantity of care (how much work is done*),* consistency of care (the understandable sequence of steps that can be followed in doing the work), coordination (the extent to which the various interdependent parts (or staff) of an organization function each according to the needs and requirements of the other parts and of the total system), and care practices (the degree to which the norms of an organization are performed).	This is associated with quality (restraints, catheter use, inadequate pain management, pressure ulcers).
14	Castle and Engberg, 2008b	Agency nurses	Organisation	If a nurse staffing model in a nursing home is used, and they use an additional regular RN per 100 beds,		Then this does not significantly improve the quality measurement (among others, physical restraint use, urinary tract infection, delirium, pressure sores, etc.).
				If a nurse staffing model in a nursing home is used, and they use an additional agency RN per 100 beds,	Then, agency RNs who work for agencies may bring innovative ideas acquired on other assignments. Alternatively, agency RNs may be highly qualified and trained and prefer the flexibility of agency work.	Then this is associated with higher overall quality.

	Included studies	Temporary nurse category	Data aggregation level	Context	Mechanism	Outcome
15	Castle et al., 2008	Nurse Aide Agency staffing	Organisation	If a nurse staffing model in a nursing home is used in which they use high levels of NA who work for agencies,		This is associated with overall low quality of care, and specifically with an increase in restraint use, an increase in catheter use, an increase in inadequate pain management and an increase in pressure ulcers.
16	Castle, 2009a	Agency staff	Organisation	If a nurse staffing model (in nursing homes) makes high use of agency staff, who are unfamiliar with the context,	Then, there is increased supervision needed from permanent nurses, which leads to higher workloads for permanent nurses.	
					Then, nursing homes pay extra for the convenience of a quick staffing fix when they are short-staffed	
17	Castle, 2009b	Agency staff	Organisation	If a nurse staffing model in a nursing home uses no agency staff,		Then this is associated with better quality.
				If a nurse staffing model in a nursing home uses higher levels of agency staff,		Then this is associated with worse quality.
18	Dall’Ora et al., 2020	Temporary RN staffing		If a nurse staffing plan is used on an adult medical and surgical hospital unit where patients are exposed to more than 1.5 h of temporary RN staffing,	RN teams are being more adaptable and able to maintain patient safety even if some staff are unfamiliar with unit practices and patients, until it reaches very high levels.	This is associated with a 12% increased hazard of death for patients.
				If a nurse staffing plan is used on an adult medical and surgical hospital unit where patients are exposed to temporary NA staffing at any level (0.5 HPPD -> 1.5HPPD),		This is associated with an increased risk of patients dying, although the effect of high levels of temporary NA staffing was much smaller than that of RNs.
19	Djukanovic et al., 2022	Agency nurse	Individual	If a nurse staffing model is used in a hospital setting (with high patient turnover), where agency nurses are used (to fill staffing gaps),	Then, agency nurses perceive themselves as competent, experienced, and committed to working closely with the patient (and being the most experienced nurse on a ward), which contributes to continuity. e.g., agency nurses could take on a leadership role.	

	Included studies	Temporary nurse category	Data aggregation level	Context	Mechanism	Outcome
					Then, agency nurses need a thorough introduction in preparation for work in a new workplace, including knowledge about co-workers’ competence, to be able to be the carrier of continuity.	
					Then, working intensively during an assignment affects continuity positively by providing better conditions for ANs and regular staff to improve teamwork	
					Gaining trust from co-workers is important to maintain continuity	
20	Fagefors et al., 2022	Staff pool	Regional	If a nurse staffing model is used in a region-wide hospital setting where they make use of a staff pool (that can be allocated to parts of the system where the existing workload and demand capacity are unusually high)	Then, qualified nurses are less willing to work in the pool to become a reliable facility in the healthcare system. The inability to staff the pool leads to inadequate service levels. Therefore, recruitment and retention of qualified staff are the main challenges for staff pool managers.	
					Then, professional competence and knowledge are needed to work on certain units.	
					The same mechanism, but reversed: then a lack of standardization, unit routines, variation in IT solutions, unstandardised documentation, and patient equipment make it more difficult for pool nurses to rotate between different clinical units in a healthcare system.	
21	Ferguson et al., 2020	Contract nurse	Unit	If a nurse staffing model is used in a large hospital that combines a high percentage of contract nurses with a high percentage of medical patients,		This is associated with higher HAPI prevalence and incidence.

	Included studies	Temporary nurse category	Data aggregation level	Context	Mechanism	Outcome
22	Gahrmann and Klumb, 2024	Temporary nurses (per diem nurses/ travel nurses)	Individual	If a nurse staffing model is used in which they make frequent use of temporary nurses (per diem nurses/travel nurses),	Then, supervising temporary nurses is perceived as unnecessary because permanent nurses resent the decision to employ temporary nurses, wishing the hospital would instead focus on retaining and recruiting permanent nurses.	Is associated with lower affective organisational commitment
					Then, supervising temporary nurses is perceived as inefficient because they constantly meet new temporary nurses.	
					Then, supervising temporary nurses is perceived as unreasonable because this may be inherent to their role, but as these tasks add up over time, it may no longer be seen as part of their role.	
23	Gan, 2022	Travel nurses/ agency nurses	Individual	If a nurse staffing model is used in which travel nurses are employed who are unfamiliar with the unit but receive higher remuneration than permanent nurses,	Then, feelings of (benign and malicious) envy may arise. Consequences of malicious envy may be communicated interpersonally, as enviers behave hostile manner towards agency nurses and gossip about envied nurses to cast doubt on other nurses’ success.	
24	Gan, 2024	Agency nurses	Individual	If a nurse staffing model is used in which agency nurses are employed who are unfamiliar on a unit,	Then, agency nurses feel a unique degree of autonomy and retain control over how they live and organise their lives,	That mitigate stress or burnout from working in permanent positions.
					Then, working as an agency nurse allows them to observe and learn from other nurses at other facilities.	
					Then, working as an agency nurse will give the nurse the freedom to focus on the clinical aspects of their work that are perceived as meaningful, without necessarily having to assume additional responsibilities typically shouldered by permanent nurses	
25	Gnanlet et al., 2023	Float nurses	Organisation	If a nurse staffing model in a hospital is used where they make lower use of floating, which is the amount of floating of nurses to units other than their home units,	Then, workers engage in varied tasks and exchange ideas among departments, and consequently, they can perform tasks more efficiently in their home department.	This positively impacts cost outcomes of the home unit (cost-savings), but to a certain threshold.

	Included studies	Temporary nurse category	Data aggregation level	Context	Mechanism	Outcome
				If a nurse staffing model in a hospital is used where they make higher use of floating, which is the amount of floating of nurses to units other than their home units,		Then this is associated with higher overall costs.
26	Griffiths et al., 2021	Flexible staff	Unit	If a staffing plan is used (based on the Safer Nursing Care Tool) with lower baseline staffing,	Many shifts were left critically understaffed as staff may not be available.	Consequently, there was a 1.7% increase in the average length of hospital stay and an 8.3% increase in the risk of death.
				If a staffing plan is used (based on the Safer Nursing Care Tool) with higher baseline staff (standard vs low standard + higher versus standard)	Many shifts were left critically understaffed as staff may not be available,	Consequently, there was less cost-effectiveness as the strategy was associated with reduced staff costs, but also with worse outcomes because the achieved staffing levels were lower, despite the use of flexible deployment.
				If a staffing plan is used (based on the Safer Nursing Care Tool) with higher baseline staff (standard vs low standard + higher versus standard)		Then it was associated with higher costs but shorter lengths of stay and fewer deaths, and became more cost effective as temporary staffing availability increased.
				If a staffing plan is used (based on the Safer Nursing Care Tool) with higher baseline staff,		
27	Griffiths et al., 2024	Temporary staff (bank or agency staff)	Organisation	If a nurse staffing model is used with low staffing levels and they make use of higher proportions of temporary staff to fill gaps,		Then the risk of death is substantially increased. While the benefits of avoiding low staffing were greater than the harms associated with using temporary staff, particularly for RNs, risk remained elevated if temporary staff were used to fill staffing shortages.
						There is no difference in effect when using bank or agency staff.

	Included studies	Temporary nurse category	Data aggregation level	Context	Mechanism	Outcome
28	Hass et al., 2006	Agency nurses	Individual level	If a nurse staffing model on an ICU is used that frequently employs agency nurses who are unfamiliar with the context,	Then, they work with different technologies in different ICU settings and varied approaches to care, and they, therefore, require assistance from the permanent nurses during their shift. Reversed, in a familiar environment, with the equipment they are accustomed to, agency nurses felt more confident in taking far less time to undertake routine aspects of care.	This may lead to feelings of lacking confidence, insecurity and diminished self-assurance, feelings of isolation, not being part of a team.
					Then, their skills may not be fully utilised, because they are often allocated to patients with low clinical acuity, or they are not allowed to administer IV medication and thereby rely on a permanent nurse, or they infrequently receive feedback.	This may lead to deskilling (reduced skills and knowledge);
29	Hurst and Smith, 2011	Temporary staff	Unit	If a nurse staffing model is used that uses permanent staff and temporary staff (compared to using only permanent staff),	Then, temporary staff are said to be less familiar with ward policy and procedures, and likely to seek help from ward colleagues.	Then they generate more unproductive time than permanent staff only wards.
					Then ward teams, including temporary staff, spent less time with patients.	
30	Jomaa et al., 2022	Agency nurses	Unit	If a nurse staffing model on a rehabilitation unit increases the proportion of agency staff members with one,	Then agency staff have the same competencies and training as regular staff and could potentially be assigned as long-term replacements, which can increase continuity of care, knowledge of the working environment, and teamwork.	This results in a 5.8% reduction in the probability of safety incidents.
31	Jones et al., 2021	Agency nurses	Organisation	If a nurse staffing model in primary health care in a remote community frequently uses temporary nurses from employment agencies who will only work for a couple of weeks,	Then, clinic-specific factors may counter any potential negative effect of staff employment stability, such as (1) having a competent clinic manager who can manage an unstable workforce more effectively,	Then this leads to a nonsignificant effect on quality of care measures in the three measured categories: child and maternal health, chronic disease management, and preventive health activity.
					Then maybe some of the short-term nurses repeatedly return to the same clinic, thus having local knowledge and long-standing relationships with residents, which facilitate continuity of care.	

	Included studies	Temporary nurse category	Data aggregation level	Context	Mechanism	Outcome
					Then skilled agency nurses enable resident remote area nurses to undertake professional development,	
					Then the acceptability of residents is not only a function of the cultural competence of non-Aboriginal staff/ torres strait islander staff, but also as a consequence of the presence of Aboriginal staff, both clinical and non-clinical.	
32	Krzyzewski et al., 2023	Travelling clinical staff	Individual	If a nurse staffing model that frequently uses TCSs who are unfamiliar with a unit,	Then, preemployment screening and appropriate placement can help avoid decreasing the burden on permanent staff who may need to supervise the TCS.	Leading to fewer perceived safety concerns.
					Then, establishing orientation specific to the facility and unit of hire, rather than covering general topics, to become familiar with unit procedures and safety practices. (e.g. offering a unit-specific resource packet such as important phone numbers, photographs of providers, policies and procedures applicable to the unit of hire).	
					Then providing support and evaluation beyond the orientation period through socialisation, teamwork, formalised feedback and providing ongoing professional development opportunities.	
					Then, collaboration and a positive team dynamic between permanent staff and TCS; efforts can be made to learn from one another to promote trust and the ability to rely on fellow team members when providing patient care.	
33	Lien, 2023	Gig nurses	Individual	If a nurse staffing model is used in nursing homes where high levels of gig nurses are used,	Then, gig nurses earn higher wages compared to permanent nurses.	This results in a deterioration in both the quality of care and the work environment, lowering staff morale and high staff turnover.

	Included studies	Temporary nurse category	Data aggregation level	Context	Mechanism	Outcome
					Then, gig nurses possess greater control over their working conditions and flexibility; not only when searching for work but also while working.	
					Then, gig services disempower permanent nurses and their environments by providing premium wages for gig nurses and balancing the extra expense by taking in more residents. Consequently, staff nurses found themselves receiving lower salaries, having higher caseloads, working on weekends and holidays, and often providing extra services for which they were not compensated.	
					Then, gig services erode social relationships and transform the meaning of care, exacerbating already depersonalised nursing home services. Staff nurses found that amid the comings and goings of gig workers, they had difficulty providing the personalised and embodied care that they deem essential.	
34	Ma et al., 2023	Agency staff	Organisation	If a nurse staffing model in a residential aged care facility relies more on agency staff (5% threshold of total direct care time in patient care),		It significantly affects quality of care measurement outcomes, such as more complaints, missing persons, reportable assaults, more hospitalisations, and accreditation flags.
35	Manias et al., 2003	Agency nurses	Individual	If a nurse staffing model uses temporary nurses who are unfamiliar with the unit,	Then, hospital managers are concerned with maintaining adequate numbers of staff.	
					Then, agency nurses do not often experience a sense of belonging to the team or the setting.	
					Then, agency nurses feel more flexible and in control of their lives.	

	Included studies	Temporary nurse category	Data aggregation level	Context	Mechanism	Outcome
					Then, temporary nurses perceive that they enhance their clinical skills by working in different settings, but opportunities for continuing education, either through agencies or the organisations they work for, are often limited.	
36	Oliveira et al., 2023	Temporary nurses	Unit level	If a nurse staffing model is used in a psychiatric hospital, where nurses work on units that frequently deploy temporary nurses (from once a month up to several times a week),		Then, nurses who work in permanent positions had significantly higher scores regarding burnout and intention to leave the profession.
37	Owens et al., 2024	Travel nurses	Individual level	If a nurse staffing model is used in which travel nurses are used who are unfamiliar with a unit,	Then, agency nurses identify flexibility as the most significant factor when choosing to travel.	
					Then, a supportive work environment is the main reason keeping them in an assignment and, in some cases, extending that assignment. Feeling as if they were welcomed by the staff and could ask questions or get help when needed proved important not only for their adjustment to the unit but also for their ability to manage the patient assignment.	
					Then, the onboarding process and the unit safety are conditions that could influence their decision to take a specific assignment or extend an existing contract. The onboarding process involves credentialing and familiarisation with regulatory requirements, which may be completed virtually before the nurse even arrives at the hospital. Completing these requirements ahead of starting would allow for unit orientation to be focused on gaining comfort within the environment	
					and building the staff relationships necessary for the travel nurse to feel supported.	

	Included studies	Temporary nurse category	Data aggregation level	Context	Mechanism	Outcome
38	Peerson et al., 2002	Agency nurses	Organisation	If a nurse staffing model frequently uses agency nurses	Then, agency nurses are sometimes assigned based on the timing of demands rather than qualifications and experience.	
					Then, hospitals frequently have preferred-provider relationships with agencies.	
					Then, hospitals regularly have policies for agency nurses, such as checking agency nurses’ practice certifications, employment conditions, orientation to the institution, length of shift and handling of incidents involving agency nurses.	
					Then, the hospital provides feedback to agencies about their nurses' performance, but the mechanism for this feedback is often informal and unstructured.	
					Then, agencies provide education for their nurse employees. Education sessions, however, are often limited to emergency procedures and do not necessarily address the long-term needs of casual nursing employees.	
39	Pham et al., 2011	Temporary staff	Organisation	If a staffing model in an emergency department frequently deploys temporary staff who are unfamiliar with the unit,	They might be unfamiliar with local staff, care management systems, protocols, and procedures. Consequently, they may face challenges in communication and teamwork, struggle to retrieve medical information, and lack clarity on which procedures to follow.	This is associated with medication errors that are more harmful than those that are associated with permanent staff.
					Temporary nurses may lag behind the latest knowledge because they typically self-manage continuing education and are usually not included in hospital continuing education services.	
					Temporary nurses may lag behind the latest knowledge because they typically self-manage continuing education and are usually not included in hospital continuing education services.	

	Included studies	Temporary nurse category	Data aggregation level	Context	Mechanism	Outcome
40	Polancich et al., 2023	Travel nurses	Organisation	If a nurse staffing model in a hospital is used with permanent staff and travel nurses,	Then, adequate nurse staffing ratios reached by a mix of active (active = permanent organisational RN staff) and travel nurses (= contracted RN staff) appear to mitigate the risk of poor patient outcomes.	This model is associated with an increase in average length of stay but not with clinical nurse-sensitive outcomes (such as CAUTIs, CLABSIs, and falls).
41	Pradhan et al., 2024	Agency staff	Organisation	If a nurse staffing model is used in a hospital in which agency staff is utilised and adequate staffing levels can be directly maintained,	Then hospitals can continue to admit and treat patients, and hospitals can respond to vagaries of patient census without long-term commitment involved in hiring permanent staff, and preventing revenue leakages.	This is associated with increased net patient revenue and increased operating revenue per bed.
					Then, agency staff wages are typically higher compared to permanent employees’ wages. Moreover, it includes additional fees for agency services and onboarding/training/monitoring costs.	This is associated with higher operating expenses. However, these higher costs may be justifiable in the short term as they may allow hospitals to provide uninterrupted patient care.
42	Saville et al., 2020	Bank/agency staff	Unit	If a nurse staffing model is used in units based on ‘low baseline’ or ‘standard baseline staffing’, then the first step is to redeploy staff between wards within the same speciality-specific division. If the shortage persists, internal bank and then external agency staff are requested. However, a request for bank or agency staff to be fulfilled on short notice is less than 50%.	Under low baseline staffing levels, units rarely have surplus staff to redeploy elsewhere.	Put patients at risk for low-quality care.
					Then this strategy may lead to unfilled shifts.	Strategy is less costly than high baseline strategy due to unfilled shifts.
					Bank or agency staff are considered less productive.	

	Included studies	Temporary nurse category	Data aggregation level	Context	Mechanism	Outcome
43	Senek et al., 2020	Agency nurses	Unit	If a nurse staffing model is used with 0 (no understaffing), and the proportion of agency nurses increases,		Then it is associated with an increased odds of ‘nursing care left undone’. The increase is most apparent in this category.
				If a nurse staffing model is used with 0.01–0.24 (low level of understaffing) and the proportion of agency nurses increases,		It is associated with increased odds of ‘nursing care left undone.
				If a nurse staffing model is used with 25% understaffing or more,	Then, agency nurses may ameliorate (but not reverse) the propensity for care left to be undone.	It is not associated with increased odds of ‘nursing care left undone’.
44	Simpson and Simpson, 2019	Agency nurses	Individual		Agency nurses feel like outsiders at units and teams, marginalised and not furnished with adequate information about the patients to whom they were assigned.	
					Agency nurses were being resented, were assumed to lack competencies until proven otherwise. (The dominant belief that the full-time standard nurse is the 'ideal nurse' further marginalised those who chose alternative work patterns.)	
					Agency nurses are denied opportunities for in-house workplace training, perpetuating a lack of career opportunities, resulting in further marginalisation as they are not equally equipped for the job. Complaints of poor educational opportunities were raised, as well as a lack of any professional feedback.	
					Agency nurses enjoyed not having to become involved in 'office politics' and disagreements about work schedules.	
					Agency nurses perceive that they are more flexible and in control of their work-life balance than traditional permanent nurses.	

	Included studies	Temporary nurse category	Data aggregation level	Context	Mechanism	Outcome
45	Twigg et al., 2016**			If a nurse staffing model is used in a small rural hospital environment, which is geographically isolated, with limited medical, clinical, and administrative support services, and they make use of temporary nurses,	Then nurses work in multiple areas of activity (ER, GP, Outpatients, Inpatient, Residential aged care, non-clinical activities);	Leads to a high workload.
					Then they are balancing the diverse workload generated by nursing activities with limited staffing resources.	
					Then the process of nurse recruitment and training is challenging: as nurses often not have the ED clinical experience needed, and therefore it requires prolonged preparation on-site to acquire the requisite clinical skills for nursing in a small rural hospital.	
46	Vander Weerdt et al., 2023	Travel nurses	Unit	If a nurse staffing model is used in which travel nurses are used to stabilise the staffing levels,	Then, open communication and collaboration can take place.	Leading to quality of care.
				If a nurse staffing model is used in which travel nurses are used to stabilise staffing levels, but the use of travel nurses is high,	Then, it is essential for managers and administrators to critically assess the work environment or the hospital unit to identify any systemic underlying conditions that may affect staffing strategy.	To protect patient care.
47	Xue et al., 2012b	Supplemental Registered Nurses	Unit	If a staffing model is used where a specific type of SRNs is employed (who were hired from several preferred staffing agencies and had a 3-month renewable contract),	Then, the policy was that experienced SRNs who had worked in similar hospital settings were preferred for hiring.	Then, SRNs are not linked to either adverse or positive patient outcomes.
					Each SRN was provided a 3-day unit-level orientation.	
48	Xue et al., 2015	Supplemental registered nurses	Unit	When the use of SRN was more than 0–0.2 or less hours per patient-day (compared with no supplemental RN overtime use).	Then, using SRNs to a certain degree could be a financial gain to hospitals compared with hiring more permanent nurses.	The average overall nursing personnel cost per patient day was decreased by $6.03 per patient day (significant difference).
				When use of SRNs was more than 0.2–0.4 or less hours per patient-day,		This was not significantly different from no use of SRNs.
				When the use of SRNs is at a level above 0.4 h per patient-day,		This was significantly related to an increase in nursing personnel costs.
49	Zaranko et al., 2022	Bank RN, Agency RN	Unit	If the proportion of rostered RN hours worked by permanent and bank RNs is increased by one percentage point,	Then, firm-specific human capital is more available in the unit.	Leading to a significant reduction of 1.2%−1.4% in the odds of patient death.
				Teams with RN staffing 16 h or more below target (resp. 20 h/ resp. 24 h or more below target),		Leading to 22.8% higher odds of patient death (resp. 26.3%/ resp. 37.0%).

Note. *Data aggregation level refers to the degree of detail at which data are analysed and reported. **The generated context-mechanism-outcome configuration derived from Twigg et al. (2016) was deemed too context-specific and therefore not taken into account in both the Tested Programme Theory and Rival Programme Theory.

AN (agency nurse); CCN (critical care nurse); CLABSI (central line-associated bloodstream infections); CLAUTI (catheter-associated urinary tract infection); ER (emergency room); GP (general practitioner); HAPI (hospital-acquired pressure injury); HPPD (hour per patient-day); ICU (intensive care unit); LPN (licensed practical nurse); NA (nurse assistant); RN (registered nurse); SRN (supplemental registered nurse); TCS (travelling clinical staff); TRNS (temporary registered nurse staffing); VAP (ventilator-associated pneumonia).

### 3.4 Tested programme theory

Some of the evidence substantiated the initial programme theory and contributed to what we further describe as the tested programme theory. We began by delineating the contexts that may trigger various mechanisms and subsequently produce outcomes.

#### 3.4.1 Context

Part of the evidence supported the described context in the initial programme theory, which is contingent upon whether a nurse staffing model is considered chronically ‘low baseline staffed’ (Griffiths et al., 2021; Saville et al., 2021; Senek et al., 2020), ‘understaffed’ (Baker et al., 2019), or if high proportions of temporary nurses in a nurse staffing model are deployed (Dall’Ora et al., 2020; Ma et al., 2023; Bae et al., 2010). According to a qualitative research study by Baker et al. (2019) in a mental health services setting, understaffing refers not only to inadequate staff numbers but also to the inappropriate distribution of skills and experience. If a nurse staffing model is considered ‘low baseline staffed’ or ‘understaffed’, temporary nurses, who are lower in familiarity, are deployed to fill staffing gaps (Saville et al., 2021; Griffiths et al., 2021; Dall’Ora et al., 2020; Ma et al., 2023; Bae et al., 2010). Low familiarity refers to the temporary nurse's unknown skills, their limited knowledge of the unit’s processes and procedures, and their unfamiliarity with the department's layout (Bajorek and Guest, 2019). In addition, low familiarity is perceived as less apparent in temporary staff from their own organisations, such as float nurses (nurses redeployed between units within the same speciality-specific division) or internal bank staff (Griffiths et al., 2021; Saville et al., 2021) but perceived as more apparent in external staff, such as agency nurses (Saville et al., 2021; Bajorek and Guest, 2019; Lien, 2023).

However, a more nuanced understanding emerges when considering the individual experience of temporary nurses. The time spent in specific roles, services, or teams and their knowledge about individual patients can be considered a mitigating factor for familiarity (Baker et al., 2019). The temporary nurse’s competencies should therefore meet the unit’s requirements (Birmingham et al., 2019). Yet, in practice, managers are primarily concerned with maintaining staff levels, and they first and foremost focus on staffing numbers and not on fit (Manias et al., 2003). Moreover, the timing of deployment was determined as important through the synthesis of the findings, yet low baseline staffing models are based on the assumption that temporary nurses can be mobilised without delay and are readily available. If a staffing model uses low baseline staffing, where surplus staff is rarely available because the call for temporary nurses to work is on short notice or in times of nursing shortages, then the demand remains unmet and will result in a staffing model that is even more critically understaffed (Saville et al., 2021; Griffiths et al., 2021). In addition, at times of peak demand, temporary nurses are occasionally assigned to work in areas where they do not have specific competencies and skills (Peerson et al., 2002) and are asked to work beyond their scope of practice (Birmingham et al., 2019). While such practices address the shortfall in staffing numbers, it was reported as coming at the cost of nurses working in teams, contributing to the perceived burden of nurses’ workload (Baker et al., 2019).

#### 3.4.2 Mechanisms

These contextual dynamics may activate various mechanisms that are observable at the unit or team level. One mechanism is the limited orientation that temporary nurses may receive at the start of their assignment in a specific clinical area (Birmingham et al., 2019). Unit routines are frequently unstandardised due to variation in documentation systems, information technology solutions, and patient equipment, as well as insufficient formalization of procedures (Fagefors et al., 2022). Researchers conducting integrative and scoping reviews have found that, without adequate patient-specific information about their assigned patients, temporary nurses feel underprepared, which can undermine confidence (Birmingham et al., 2019; Simpson and Simpson, 2019). As a result, they rely on permanent nurses for clinical information and guidance because of the lack of standardised unit routines (Birmingham et al., 2019; Hurst and Smith, 2011; Hass, 2006). This unfamiliarity and dependency can lead to slower and less efficient work (Saville et al., 2021; Bajorek and Guest, 2019), increasing the workload burden on permanent nurses, who are implicitly expected to provide supervision and feedback – tasks that distract them from their own responsibilities, takes them away from their patients, and results in rushed activities (Bajorek and Guest, 2019; Berg Jansson and Engström, 2017; Castle, 2009; Hurst and Smith, 2011). Due to their reliance on permanent nurses, temporary nurses may feel underutilised. For example, they are frequently assigned to patients with low clinical acuity, or they may not be permitted to administer intravenous medication (Hass, 2006).

A further mechanism that could be activated in response to the practical burden is that permanent nurses may perceive their supervising role as unfair, particularly when there is underlying resentment toward the employment of temporary staff (Gahrmann and Klumb, 2024). Although guiding and supervising newcomers is part of their professional role, the cumulative burden can lead to feelings of devaluation (Gahrmann and Klumb, 2024). Such tensions are likely to intensify when temporary nurses earn considerably higher wages than permanent staff, potentially violating key workplace norms and provoking envy, resentment, or exclusionary behaviours (Gan, 2022; Lien, 2023).

Another mechanism that can be triggered relates to the erosion of collaborative team processes under conditions of low staffing levels and high workload. Collaboration and help-seeking behaviours tend to flourish in units with stable work relationships but are less common in those that depend heavily on temporary nurses (Bae et al., 2010). These units often face increased workload pressures (Baker et al., 2019). Although effective communication is essential for teamwork and strong work relationships, it tends to deteriorate under such conditions, ultimately compromising patient care (Birmingham et al., 2019) and service delivery (Bajorek and Guest, 2019).

A distinct mechanism that could be activated pertains to the limited formalised learning opportunities available to temporary nurses. Although they may receive informal on-the-job feedback during shifts, access to performance-related feedback is inconsistently implemented (Pham et al., 2011; Peerson et al., 2002; Manias et al., 2003). While organisations may provide feedback to the external agencies that employ temporary staff, these feedback loops are typically unstructured, which limits their effectiveness. Additionally, researchers conducting a scoping review found that opportunities for formal, continuous learning were scarce (Simpson and Simpson, 2019) and failed to address the long-term learning needs of temporary nurses (Peerson et al., 2002). For instance, whilst permanent nurses attend educational sessions, the temporary nurses are expected to continue patient care delivery (Birmingham et al., 2019).

The last mechanism that illuminated was, according to Simpson & Simpson (2019), an enduring and largely implicit belief that persists within the nursing profession that the ideal nurse is a permanent, preferably full-time, staff member, while temporary nurses are viewed as subordinate. This belief reinforces a dualistic conceptualisation of the nursing workforce, distinguishing between ‘core’ and ‘peripheral’ staff (Gan, 2024). Permanent nurses are highly valued for their familiarity with unit routines and their consistent contributions to continuity of care. In contrast, temporary nurses are perceived as lacking these qualities. Moreover, because temporary nurses are often allocated to patients with lower complexity, they may not fully utilise their potential, potentially leading to deskilling or feelings of insecurity (Hass et al., 2006). These dynamics further contribute to the temporary nurses’ experiences of marginalisation, isolation, and lack of emotional support within the team (Simpson and Simpson, 2019; Jamieson et al., 2008; Manias et al., 2003). In turn, this can disrupt communication at multiple levels of care delivery (Batch and Windsor, 2015). In response to this pervasive perception, newly arrived individual temporary nurses can feel pressured to demonstrate their competence and prove their worth (Simpson and Simpson, 2019; Gan, 2022).

#### 3.4.3 Outcomes

The activated mechanisms may produce outcomes for patients, nurses, and health systems. Deploying higher proportions of temporary nurses in hospitals with low staffing levels was associated with adverse patient outcomes, such as an increased risk of patient falls (Bae et al., 2014a, 2014b), pressure ulcers (Ferguson et al., 2020), an increase in average hospital stay length (Griffiths et al., 2021), and a higher risk of patient death (Griffiths et al., 2024, 2021; Dall’Ora et al., 2020; Zaranko et al., 2022). Furthermore, when a nurse staffing model in a hospital was only slightly understaffed and the proportion of agency nurses increased, this was associated with increased odds of ‘nursing care left undone’ (Senek et al., 2020). In contrast, in cases of severe understaffing, the deployment of temporary nurses may improve, but not eliminate, the likelihood of care being left undone, as this was not associated with an increased risk of ‘nursing care left undone’ (Senek et al., 2020). If understaffing in a mental health service setting becomes a regularity with greater reliance on temporary staff, it may lead to waiting lists for patients and less effective treatment, consequently leading to more completed patient suicides (Baker et al., 2019). Additionally, reliance on agency staff in residential care institutions and nursing homes may significantly impact the quality of care (Bourbonniere, 2006), such as increases in restraints, catheter use, inadequate pain management, and pressure ulcers (Castle and Engberg, 2008a; Castle et al., 2008b; Castle, 2009); more complaints, more hospitalizations, and an increase in the number of unmet quality standards identified by the Aged Care and Quality Safety Commission in accreditation inspections (Ma et al., 2023). This becomes even more pronounced when high levels of temporary nursing aides are deployed (Castle et al., 2008). In the worst case, the employment of large numbers of temporary nurses can transform the meaning of care, further exacerbating the already depersonalised nature of nursing home services and ultimately contributing to a decline in both quality of care and the work environment (Lien, 2023).

In support of the tested programme theory, several researchers also indicated unfavourable outcomes for nurses, such as increased reporting of burnout (Oliveira et al., 2023), higher staff sickness rates (Baker et al., 2019), back injuries (Bae et al., 2010), lower affective organisational commitment (Gahrmann and Klumb, 2024), and recruitment and retention problems (Baker et al., 2019; Oliveira et al., 2023). Additionally, a high proportion of temporary nurses has been linked to increased nursing personnel costs (Xue et al., 2015; Gnanlet et al., 2023).

### 3.5 Rival programme theory

We identified context-mechanism-outcomes configurations that refuted the initial programme theory and generated a rival programme theory. While the tested programme theory posits that temporary nurses are subordinate to permanent nurses and are primarily used to fill staffing gaps, the rival programme theory posits that temporary nurses are equally valuable and complementary to permanent staff.

#### 3.5.1 Context

The rival programme theory suggests that a nurse staffing model should focus on achieving an adequate match between the demand for nursing work and available resources. In this model, temporary nurses should be used to stabilise a nurse staffing plan, as indicated by a systematic review (Vander Weerdt et al., 2023). Temporary nurses possess competencies and training equivalent to permanent nurses and are generally considered to be equally, if not more, competent and experienced. This makes them suitable candidates for long-term assignments (Jomaa et al., 2022; Djukanovic et al., 2022) and means that there is little difference between the two groups (Aiken et al., 2007). Compared to permanent staff, temporary nurses have less knowledge about contextual care routines and implicit organisational practices and relationships, which are perceived as essential for ensuring continuity of care (Baker et al., 2019; Djukanovic et al., 2022; Castle and Engberg, 2008a). Therefore, if the proportion of temporary nurses increases while an adequate number of permanent nurses is maintained, the necessary knowledge is likely to remain widely accessible within the department (Zaranko et al., 2022) and may not result in the burden described in the tested theory. Moreover, temporary nurses may also bring in innovative ideas acquired on other assignments (Berg Jansson and Engström, 2017; Castle and Engberg, 2008b).

More importantly, the work environment has consistently emerged as a critical contextual factor shaping both the employment of temporary nurses and the related quality outcomes (Jones et al., 2021; Owens et al., 2024). Multiple researchers (Aiken et al., 2013; Bae et al., 2014a) have suggested that the association between greater reliance on temporary nurses and adverse patient outcomes is largely attributable to suboptimal work environments; i.e., work environments that are structured and shaped to fit permanent staff’s needs, rather than those of temporary nurses. These contextual conditions about how the work environment is shaped activate a series of potential mechanisms.

#### 3.5.2 Mechanisms

Several mechanisms are triggered to safeguard and strengthen continuity of care processes and collaboration. One mechanism that supports continuity of care involves implementing comprehensive unit-specific orientation programmes, onboarding, and prioritizing the deployment of temporary nurses with prior experience in similar settings (Xue et al., 2012b; Dall’Ora et al., 2020; Djukanovic et al., 2022; Owens et al., 2024; Berg Jansson and Engström, 2017). This helps adapt and equip teams, while also reducing the burden on permanent staff responsible for supervising temporary nurses (Krzyzewski et al., 2023). Another mechanism that can be activated was described in an integrative review as cultivating collaboration and open communication between temporary and permanent nurses, which contributes to positive team dynamics (Krzyzewski et al., 2023). It fosters a sense of trust, enabling permanent and temporary nurses to rely on and learn from one another (Vander Weerdt et al., 2023; Krzyzewski et al., 2023). This mutual trust facilitates real-time on-the-spot feedback that reinforces a sense of joint teamwork and continuity of care (Jomaa et al., 2022; Djukanovic et al., 2022; Bae et al., 2010).

Temporary nurses recognizing and heeding their lack of embedded knowledge can activate another mechanism, namely that they adopt a more reflective, cautious, and questioning approach. This may enhance learning, strengthen collaboration and care continuity and enable them to play the role of reflective practitioner (Bae et al., 2010; Gan, 2024) or take on a leadership role (Djukanovic et al., 2022).

Furthermore, some mechanisms that can be activated specifically benefit temporary nurses. By choosing to work as a temporary nurse, they may experience a unique sense of autonomy and control over their work-live balance, which gives them the flexibility to especially focus on the clinical aspects of their work that are perceived as meaningful by them (Gan, 2024; Lien, 2023; Owens et al., 2024; Berg Jansson and Engström, 2017). In addition, working across various healthcare contexts allows them to observe different practices and learning opportunities that contribute to their professional development (Gan, 2024).

#### 3.5.3 Outcomes

As a result of the activated mechanisms, the rival programme theory encompasses favourable patient outcomes, such as fewer medication errors (Bae et al., 2010), a reduced probability of safety incidents (Jomaa et al., 2022), reduced length of stay (Griffiths et al., 2021), and a reduced risk of patient deaths (Griffiths et al., 2021; Dall’Ora et al., 2020), and higher overall care quality in nursing homes (Castle and Engberg, 2008a). Moreover, adequate staffing levels indicate higher proportions of staff with embedded knowledge, such as permanent nurses, which reduces pressure ulcers and physical restraints (Castle and Engberg, 2008b). Although the employment of temporary nurses may increase operating costs due to higher wages, agency fees, and onboarding costs, it enables hospitals to avoid low staffing levels that could compromise patient care quality, ultimately increasing both net patient revenue and operating revenue per bed (Pradhan et al., 2024).

### 3.6 Synthesis

**Fig. 2:**
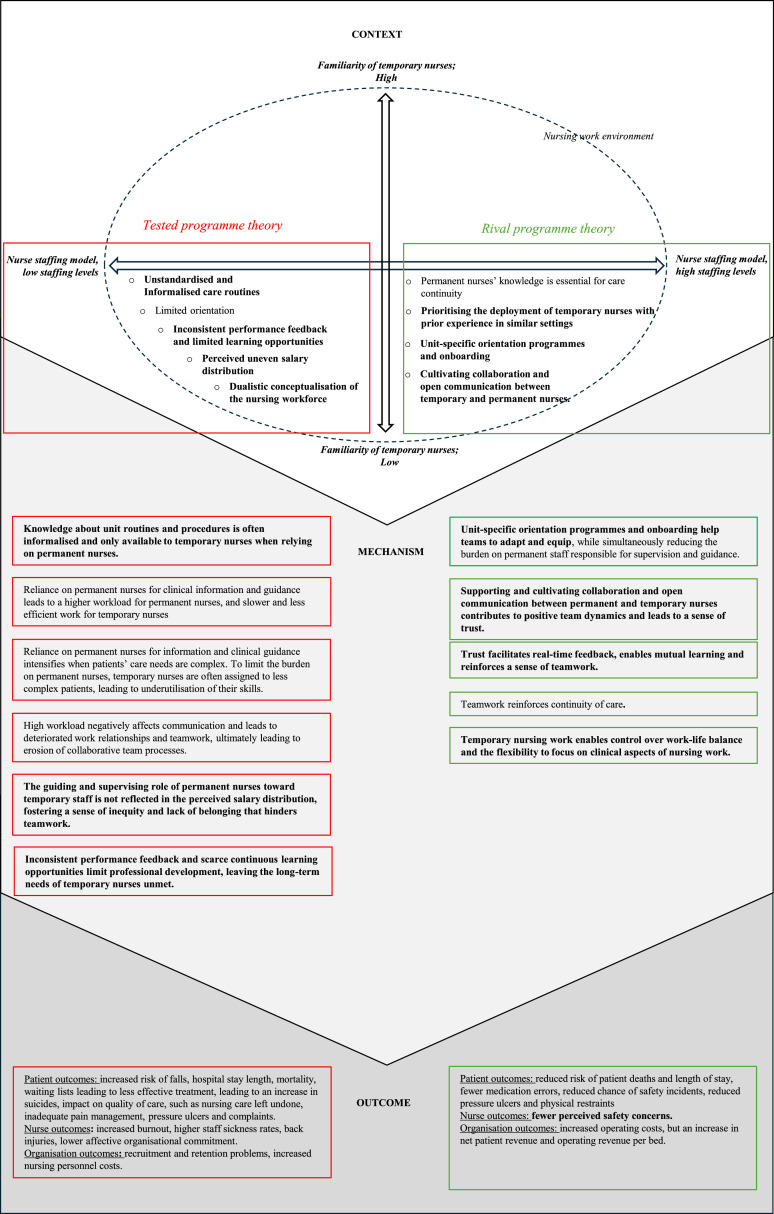
General model incorporating the tested and the rival programme theory. Note: Red boxes indicate components of the tested programme theory. Green boxes denote components of the rival programme theory. Text presented in bold highlights elements of the programme theories derived from included manuscripts considered of high quality.

To synthesise the identified contexts, mechanisms, and outcomes, a general model was drafted, incorporating the tested and rival programme theories (Fig. 2). The model illustrates how contextual factors, such as staffing levels and (un)familiarity of temporary nurses, activate mechanisms at different layers of reality, leading to favourable or unfavourable outcomes for patients, nurses, and organisations. In the tested theory, high proportions of temporary nurses combined with low staffing levels may activate multiple mechanisms. At the surface level, temporary nurses rely on permanent nurses for clinical information and guidance. Over time, this may elicit feelings of unfairness and devaluation among permanent nurses, possibly contributing to outcomes such as retention problems. Simultaneously, increased reliance may heighten workload pressure, which potentially inhibits team collaboration (e.g., help-seeking behaviours) and potentially compromises patient care and service delivery.

The rival theory suggests that temporary nurses can stabilise staffing models when sufficient permanent staff are retained, ensuring broad access to knowledge. Several researchers also identify the work environment as a key contextual factor, as it is often organised for permanent rather than unfamiliar staff. This may trigger various mechanisms at different layers. At the surface, unit-specific orientation programmes may improve temporary nurses’ knowledge and team preparedness. At a deeper relational level, this may foster more equal dynamics between permanent and temporary staff, enabling open communication and collaboration and potentially resulting in fewer incidents and higher quality of care.

## 4 Discussion

### 4.1 Evidence synthesis and programme theory development

This realist review tested an initial programme theory on nurse staffing models that frequently employ temporary nurses unfamiliar with the unit to examine through which mechanisms these models generate patient, staff, and health system outcomes in particular contexts. Evidence emerged that supported the initial programme theory but also generated a rival programme theory. Together, the theories help explain the mixed findings of previous studies on the effects of employing temporary nurses. The tested programme theory indicates that heavy reliance on temporary nurses in settings with low baseline staffing levels can activate several mechanisms leading to unfavourable outcomes. These include dependency on permanent nurses for guidance and supervision, lack of standardised work practices, inappropriate skill distribution, limited patient-specific knowledge, unstable teamwork, and suboptimal communication, which may disrupt care continuity and compromise care delivery. Such mechanisms can generate adverse patient outcomes and undesirable outcomes for nurses, contributing to recruitment and retention challenges. Moreover, low staffing levels combined with extensive use of temporary nurses may activate mechanisms across multiple layers of reality, potentially eroding the essence of nursing care and undermining care quality and the work environment. The rival programme theory further suggests that high deployment of temporary nurses reflects a poor work environment.

The tested programme theory aligns with Griffiths et al. (2024) and Dall’Ora et al. (2022), showing that low staffing levels and poor team composition are linked to increased mortality. The rival programme theory resonates with the work of Marufu et al. (2021), highlighting how poor work environments exacerbate staffing issues and increased reliance on temporary staff. Given the global nursing shortage, the use of temporary nurses may be a continuing practice. Their proper integration into nurse staffing models, along with broader workforce support and retention, is essential to reduce dependence on temporary nurses (Wallenburg et al., 2023; Dingelstad et al., 2024).

The rival programme theory identifies an often-overlooked mechanism: the belief that permanent full-time nurses represent the ideal or ‘gold standard’. Rooted in traditional notions of the ‘good nurse’, this ideal values stability and familiarity as the basis of quality care (Fealy, 2004; Van der Elst, 2012). Continuity of care is thus associated with stable, long-term employment rather than fluid, short-term interactions (Haggerty et al. 2003). This theory suggests reframing temporary nurses as stabilising additions rather than merely gap-fillers. Academics, policymakers, and the workforce should view temporary nurses as valuable and complementary staff because they possess competencies comparable to permanent nurses and can contribute to team collaboration, mutual learning, and positive team dynamics, which possibly mitigate risks of poor patient, nurse, and organisational outcomes.

Both theories highlight that familiarity is crucial for nursing care. From a retroductive perspective, the latent mechanism underneath familiarity may be the tacit, situated knowledge of the organisation and patients, mostly held by permanent nurses (Herbig et al., 2001). Temporary nurses often rely on this knowledge, requiring frequent communication and close collaboration (Nonaka et al., 1996). Schmelzer et al. (2025) showed that permanent nurses recognise this knowledge and adapt their communication to bridge the gap, using explicit and elaborated language to support temporary understanding.

As the nursing shortage intensifies and employment shifts toward short-term contracts and an emphasis on work-life balance, healthcare employers should remain attentive to how crucial tacit knowledge is embedded and accessed in daily practice. From our realist review, we have demonstrated that unfamiliar temporary nurses depend on permanent staff to access the situated knowledge about the organisation and patients necessary for care provision. Echoing McNamara and Fealy (2010), this reveals a structural issue in which permanent nurses compensate for systemic deficiencies, reflecting suboptimal work environments (De Kok, 2023). Healthcare organisations should acknowledge the absence of such knowledge among unfamiliar temporary nurses and create environments that make it more tangible and visible, supporting both permanent and temporary staff.

The nursing work environment encompasses organisational characteristics that facilitate or constrain professional nursing practice, including staffing; resources; collegial nurse-physician relationships; managers’ ability; leadership and support; nurse participation in hospital affairs; and nursing foundations for quality care (Lake, 2002; Swiger et al., 2017). Supportive work environments are often related to positive outcomes for patients (Stalpers et al., 2015) and nurses (Tan Jr et al., 2024; Ronnie, 2020), a finding reinforced by the rival programme theory. However, current environments often fail to enable experienced but unfamiliar temporary nurses to work effectively. Work environments that are intuitively accessible and shaped by embedded knowledge of ward routines, protocols, and patient-specific information support equitable work distribution and reduce the burden on permanent staff (Fagefors et al., 2022).

Practical strategies include unit-level induction and rapid orientation for temporary nurses, preferably on the unit level (Telschow and Berg, 2025), relieving permanent staff of supervision duties and promoting positive communication and collaboration. Standardising work routines (e.g., consistent medication room layouts, intuitive information technology systems, documentation practices, or uniform protocols further supports integration (Fagefors et al., 2022; Fadda, 2018). Recognising the value of situational knowledge is, therefore, essential for designing work environments that sustain collaboration between permanent and temporary nurses.

### 4.2 Strengths and limitations

A key strength of this realist review was the inductive development of an initial programme theory through a systematic search of the literature conducted by a multi-national team. National priorities and stakeholder engagement guided the lines of inquiry for initial programme theory testing. Another strength of this study was the use of a realist review approach, which allowed us to examine different context-mechanism-outcomes relationships. This led to the development of two distinct programme theories about the employment of temporary nurses, reflecting the ambiguous evidence concerning the association between the use of temporary staffing and patient and nurse outcomes. A further strength was the inclusion of diverse study designs following realist methodology (Wong, 2018), which allowed for robust assessment of relevance, richness, and rigour, thereby enabling the exploration of new contexts, mechanisms, and outcomes. However, several sources described different levels of data aggregation, complicating sensemaking and potentially affecting the robustness of the conclusions. Nonetheless, this offered an enriched understanding of how contexts, mechanisms, and outcomes could be at play across multiple levels across different settings. A limitation of this study was the inclusion of evidence on nurse staffing models involving temporary nurses from database inception. This decision was based on the need to identify sufficiently rich records to build a thorough understanding of the context, the underpinning potential mechanisms, and the resulting outcomes. Such comprehensiveness is particularly important in realist evaluations, as understanding the interplay between contexts, mechanisms, and outcomes forms the methodological foundation (Greenhalgh et al., 2011). However, contextual factors regarding the deployment of temporary nurses may have changed over time, especially during and after the COVID-19 pandemic. As a result, some context-mechanism-outcome configurations from older studies may be less directly applicable to current practice. Nevertheless, although current contextual relevance may differ from that in older studies, human responses to certain situations may transcend time, such as allocating temporary nurses to patients with lower clinical acuity or complexity (Hass, 2006) or the supervisory role of permanent nurses over temporary nurses (Castle et al., 2009a). Another limitation is related to the conceptualization of temporary nurses. Definitions differ across studies and contexts, thereby complicating comparisons. Nevertheless, the shared denominator of unfamiliarity and the role of implicit, tacit knowledge offered a consistent analytical lens, highlighting the contextual nature of temporary nurse employment.

### 4.3 Implications for practice

The findings of this realist review may have important implications for practice. In contrast to common practice, employers should consider emphasizing the nursing work environment by prioritizing and investing in an intuitive work environment for rapid orientation. Organisations that heavily depend on temporary nurses may be able to benefit greatly from this attention shift. Consequently, the research findings may have significant implications for nursing work, as it suggests that it enriches nursing work rather than eroding it because it prevents permanent nurses from having too much on their plate. Moreover, temporary nurses may feel welcomed and valued, consequently enabling both permanent and temporary nurses to focus on the aspects of their work that they perceive as meaningful. This may lead to a positive influence on the recruitment and retention of nurses (Seller-Boersma et al., 2023; De Vries et al., 2023).

One important practical implication is to turn the talk about permanent and temporary nurses as distinctive groups, starting with the nurses themselves. By doing so, nurses may be able to acknowledge that there is no such thing as an ‘ideal’ versus ‘subordinate’ nurse and break with this traditional view. While in organisations and legislation, temporary nurses are still viewed as marginal, these professionals are entrepreneurs who demonstrate that current staffing policies no longer align with today’s organisational healthcare realities. As staffing shortages persist, reality will force change. The rival programme theory may provide an answer by valuing both permanent and temporary nurses and point towards a supportive, intuitive work environment where all nurses, regardless of employment status, are seen as equal contributors to patient care.

## 5 Conclusion

With temporary nurses a continuing staffing presence, we have highlighted the need for more continued research on nurse staffing models in which temporary nurses are deployed. Seeing temporary nurses solely as gap fillers is limited. Instead, we recommend addressing shortages by focusing on ensuring adequate nurse staffing levels and fostering collaboration and trust between permanent and temporary nurses by investing in a supportive work environment with rapid onboarding and orientation to enhance temporary nurses’ familiarity with the often tacit knowledge of specific contexts and patients. When a more intuitive work environment is created, temporary nurses may be able to utilize their experience and expertise, giving room for mutual learning. This investment probably leads to a positive influence on the recruitment and retention of nurses.

## Funding

This study was funded by a grant from the Canadian Institutes of Health Research (#169,127) to Greta G. Cummings.

## Declaration of generative AI and AI-assisted technologies in the writing process

During the preparation of this work, the first author used Grammarly and ChatGPT in order to improve language and readability. After using this tool/service, the first author reviewed and edited the content as needed and takes full responsibility for the content of the publication.

## CRediT authorship contribution statement

**Inge Wolbers:** Writing – original draft, Visualization, Software, Methodology, Investigation, Formal analysis, Data curation, Conceptualization. **Catharina van Oostveen:** Writing – review & editing, Validation, Supervision, Methodology, Investigation, Formal analysis, Conceptualization. **Dewi Stalpers:** Writing – review & editing, Supervision, Software, Methodology, Investigation, Formal analysis, Conceptualization. **Greta G. Cummings:** Writing – review & editing, Validation, Supervision, Investigation, Funding acquisition, Conceptualization. **Kaitlyn Tate:** Writing – review & editing, Supervision, Project administration, Methodology, Funding acquisition, Conceptualization. **Lisette Schoonhoven:** Writing – review & editing, Validation, Supervision, Formal analysis. **Pieterbas Lalleman:** Writing – review & editing, Supervision, Methodology, Investigation, Formal analysis, Conceptualization.

## Declaration of competing interest

We have nothing to declare.

## Appendix Supplementary materials

Image, application 1

Image, application 2

Image, application 3
