# Supplementary material for: Nurse staffing models that rely on employment of temporary nurses: A realist review
Source: Int J Nurs Stud Adv. 2026 Apr 28;10:100537. doi: 10.1016/j.ijnsa.2026.100537 (PMC13137211; doi:10.1016/j.ijnsa.2026.100537)
Supplement: Supplementary file 1 [file mmc1.docx]

Supplementary File 1: The Search String

PUBMED:

((("bank nurs*" OR "part-time nurs*" OR "casual nurs*" OR "temporary nurs*" OR "agency nurs*" OR "per diem nurs*" OR "bank staff" OR "part-time staff" OR "casual staff" OR "temporary staff" OR "agency staff" OR "part-time employment" OR "temporary employment" OR "flexible employment" OR "supplemental nurse*" OR "floating nurse" OR "temporary hires" OR “travel nurs*”) AND (nurs*)) AND (“staffing model” OR “Personnel Staffing and Scheduling”[Mesh] OR “flex nurse staffing” OR “flex staffing”)

EMBASE:

((("bank nurs*" OR "part-time nurs*" OR "casual nurs*" OR "temporary nurs*" OR "agency nurs*" OR "per diem nurs*" OR "bank staff" OR "part-time staff" OR "casual staff" OR "temporary staff" OR "agency staff" OR "part-time employment" OR "temporary employment" OR "flexible employment" OR "supplemental nurse*" OR "floating nurse" OR "temporary hires" OR “travel nurs*”) AND (nurs*)) AND ("staffing model" OR "Personnel Staffing and Scheduling"/exp OR "flex nurse staffing" OR "flex staffing"))

CINAHL:

((("bank nurs*" OR "part-time nurs*" OR "casual nurs*" OR "temporary nurs*" OR "agency nurs*" OR "per diem nurs*" OR "bank staff" OR "part-time staff" OR "casual staff" OR "temporary staff" OR "agency staff" OR "part-time employment" OR "temporary employment" OR "flexible employment" OR "supplemental nurse" OR "floating nurse" OR "temporary hires" OR “travel nurs*”) AND (“nurs*”)) AND ( MH “Personnel Staffing and Scheduling" OR "flex nurse staffing" OR "flex staffing" OR "staffing model"))

Scopus:

TITLE-ABS-KEY ( "bank nurse" OR "part-time nurs*" OR "casual nurs*" OR "temporary nurse" OR "agency nurs*" OR "per diem nurs*" OR "bank staff" OR "part-time staff" OR "casual staff" OR "temporary staff" OR "agency staff" OR "part-time employment" OR "temporary employment" OR "flexible employment" OR "supplemental nurse" OR "floating nurse" OR "temporary hires" OR "travel nurs*" AND "nurs*" ) AND TITLE-ABS-KEY ( "staffing model" OR "Personnel Staffing and Scheduling" OR "flex nurse staffing" OR "flex staffing" )
