# Supplementary material for: Nurse staffing models that rely on employment of temporary nurses: A realist review
Source: Int J Nurs Stud Adv. 2026 Apr 28;10:100537. doi: 10.1016/j.ijnsa.2026.100537 (PMC13137211; doi:10.1016/j.ijnsa.2026.100537)
Supplement: Supplementary file 2 [file mmc2.docx]

**Supplementary File 2: Inclusion and Exclusion criteria**

INCLUSION CRITERIA

**PART A:** Must include 1 and 2 and must include either 3 or 4, or 5 or 6.

1. A nurse staffing model (which may include other healthcare professionals) conceptualised, developed, implemented or evaluated in any of the following health settings (in-patient and out-patient settings including acute care settings (ED and in hospital), primary care settings, community settings, palliative care settings, transitional or rehabilitation care settings and continuing care settings).

2. A nurse staffing model conceptualized, developed, implemented or evaluated in high-income countries based on World Bank income groups ([*https://data/worldbank.org/income-level/high-income*](https://data/worldbank.org/income-level/high-income)).

3. The main focus of the paper is to include a formal or substantive theory, mid-range theory, theoretical/conceptual framework that describes how nurse staffing models are intended to work.

4. The main focus of the paper is to review/provide ideas about how nurse staffing models are intended to work or provide a critique of the ideas underlying how nurse staffing models are intended to work.

5. The main focus of the paper is to provide stakeholder accounts or opinions of how nurse staffing models do OR do not work.

6. The main focus of the paper is to outline, discuss or review potential unintended consequences of nurse staffing models.

**PART B:** The following inclusion criterium must be included in addition to those mentioned in part A.

Empirical evidence that is relevant, rich and robust and supports, challenges or gives insight into a nurse staffing model that frequently uses non-full time and temporary nurses who are unfamiliar with a unit (context), then this threatens care continuity and permanent RN staff will feel overburdened and demotivated (mechanisms), leading to poor teamwork, burnout, more adverse events, longer hospital stays and increase costs (outcomes).

EXCLUSION CRITERIA:

1. Articles not written in English or Dutch
2. A staffing model that focuses on midwives/nurse-midwives (or other healthcare professionals) only
3. A nurse staffing model conceptualized, developed, implemented, or evaluated in any of the following health settings: academic and/or administrative settings (e.g. nurses in policy or executive management positions)
4. A nurse staffing model conceptualized, developed, implemented, or evaluated in low income, lower-middle, or upper-middle income countries
5. The main focus of the paper is to report findings of an intervention that is conducted by a nurse staff model, without examining or evaluating the staff model (e.g. a study examining an intervention of geriatric assessment/ambulation efforts to improve hospital post-discharge outcomes and is simply reported as conducted by nursing staff/multidisciplinary team).
6. Statistical models (Regression, Markov) or Models of care (integrative or integrative care) or Disease models (animals used to study human diseases). Note that we won’t exclude articles simply if these are included in the paper, but instead if they are the type of ‘model or framework’ being discussed instead of nurse staffing.
7. Articles that examine concepts closely related to nurse staffing, but do not link it to nurse staffing (i.e. staff/skills mix).
8. Conference abstracts and proceedings, news items, protocols, interviews, ‘tweets of the week’, ‘union rallies’, commentaries that describe a referenced model (label as ‘snowball’ and search for referenced model instead)
