# Supplementary material for: Nurse staffing models that rely on employment of temporary nurses: A realist review
Source: Int J Nurs Stud Adv. 2026 Apr 28;10:100537. doi: 10.1016/j.ijnsa.2026.100537 (PMC13137211; doi:10.1016/j.ijnsa.2026.100537)
Supplement: Supplementary file 3 [file mmc3.docx]

Supplementary File 3: Quality appraisal.

**Descriptions and traffic light criteria for quality appraisal on relevance, richness and rigour.**

**PART A:** Descriptions of relevance, richness and rigour

Relevance; whether the data can contribute to theory building and/or testing for included contexts/regions (i.e. the Netherlands, Australia, Canada and Great Britain).

Richness; the degree of theoretical thickness and conceptual richness that explains how an intervention is expected to work based on grounded and detailed descriptions

Rigour; the trustworthiness of the source, and coherence and transparency of the theory.

(adapted from Tate et al., 2024; Dada et al., 2023).

**PART B:** Traffic light system (adapted from Dada et al., 2024; Jagosh et al., 2011; Morton et al., 2021).

|  | Minor concern (green) | Moderate concern  (yellow) | Major concern  (red) |
| --- | --- | --- | --- |
| Relevance | Data can contribute to theory building/ or testing and is from NL, Aus, Ca or GB | Data can contribute to theory building/ or testing but is not from NL, Aus, Ca or GB | Data does not contribute to theory-building/ or testing |
| Richness  Using a score ranging from 0 to 5. | 4 = much valuable data | 2 = limited data of interest, but quick to extract it and could add weight to findings. 3 = some good quality criteria. | 0 = Nothing of interest/ not focused on design implementation or use. 1 = limited data of interest, likely to appear in other articles. |
| Rigour  Is this data likely to be biased?  Is it dealt with critically?  Is it from a real-world example or theoretical speculation?  Was the data gathered in some depth over time or in a quick ‘snapshot’? Is it safe to generalise from this data? | The questions are answered with minor concerns about trustworthiness, coherence and transparency. | The questions are answered with moderate concerns about trustworthiness, coherence and transparency. | The questions are answered with major concerns about trustworthiness, coherence and transparency. |

**Procedure for the application of the traffic light system.**

1. **Relevance is first assessed.**
2. **Assessment of richness.**
3. **A) Subsequently, the trustworthiness of the evidence is assessed.** *(Is this data likely to be biased? Is it dealt with critically? Is it from a real-world example or theoretical speculation? Was the data gathered in some depth over time or in a quick “snapshot”? Is it safe to generalise from this data?)*
4. **B) The coherence of the emerging theory is examined** (*A coherent theory is consilient* (*explains the data*), *simple* (*makes few assumptions*), *and analogous to substantive theory* (*aligns with existing credible theories*)).

**Example of how quality appraisal was performed in practice.**

The following example shows that the quality appraisal in realist review is not a linear process, and it shows how researchers applied their insights and judgments in the consensus meetings.

As an example, we consider Baker et al. (2019). This exploratory, qualitative study was first independently assessed by researchers IW and CO, after which a consensus meeting was held.

During this meeting, we discussed relevance, richness, and rigour. We both assessed the study as ‘minor’ on relevance, and as ‘moderate’ on richness (having initially scored 3), but disagreed on rigour (scoring ‘minor’ and ‘moderate’).

In a consensus meeting, we discussed the trustworthiness and the coherence of the study. As CO is an expert on nurse staffing, she provided broader contextual insights and judged that the theory proposed by Baker et al. (2019) offered a clear illustration of how low staffing levels and the use of temporary nurses could lead to adverse outcomes.

Following this meeting, we noted that we had ‘minor/moderate’ concerns about the study, mainly because of our (slight) reservations regarding richness.

However, as we moved forward in our theory-building process, we found ourselves repeatedly returning to Baker et al.'s proposed theory on the vicious cycle of unsafe staffing. In realist terms, we came to view this study as a ‘nugget of wisdom’, also because with their theory, they provided a possible explanation of why phenomenon X could lead to Y (Dada et al., 2023). Therefore, we ultimately appraised the record as minor, without any concern.

**References**

Baker, J. A., Canvin, K., & Berzins, K. (2019). The relationship between workforce characteristics and perception of quality of care in mental health: A qualitative study. *International Journal of Nursing Studies*, *100*, 103412. <https://doi.org/10.1016/j.ijnurstu.2019.103412>

Dada, S., Dalkin, S., Gilmore, B., Hunter, R., & Mukumbang, F. C. (2023). Applying and reporting relevance, richness and rigour in realist evidence appraisals: advancing key concepts in realist reviews. *Research synthesis methods, 14*(3), 504-514.

Jagosh, J., Pluye, P., Macaulay, A. C., Salsberg, J., Henderson, J., Sirett, E., … & Green, L. W. (2011) Assessing the outcomes of participatory research: protocol for identifying, selecting, appraising and synthesizing the literature for realist review. *Implementation science, 6,* 1-8.

Morton, T., Wong, G., Atkinson, T., & Brooker, D. (2021). Sustaining community-based interventions for people affected by dementia long term: the SCI-Dem realist review. *BMJ Open, 11*(7), e047789.

Tate, K., Penconek, T., Booth, A., Harvey, G., Flynn, R., Lalleman, P., Wolbers, I., Hoben, M., Estabrooks, C. A., & Cummings, G. G. (2024). Contextually appropriate nurse staffing models: a realist review protocol. *BMJ Open, 14*(5), e082883.
